# DeepSAP: an integrated generative and predictive deep-learning framework for controllable design of self-assembling peptides

**DOI:** 10.1093/bioinformatics/btag744

**Published:** 2026-10-07

**Authors:** Yuanming Song, Yuhan Wu, Yuqiu Jiang, Li He, Shoubo Zhao, Xiaoyuan Deng

**Affiliations:** MOE Key Laboratory of Laser Life Science & Institute of Laser Life Science, College of Biophotonics, School of Optoelectronic Science and Engineering, South China Normal University, Guangzhou 510631, China; Guangdong Provincial Key Laboratory of Laser Life Science, Guangzhou Key Laboratory of Spectral Analysis and Functional Probes, College of Biophotonics, School of Optoelectronic Science and Engineering, South China Normal University, Guangzhou 510631, China; MOE Key Laboratory of Laser Life Science & Institute of Laser Life Science, College of Biophotonics, School of Optoelectronic Science and Engineering, South China Normal University, Guangzhou 510631, China; Guangdong Provincial Key Laboratory of Laser Life Science, Guangzhou Key Laboratory of Spectral Analysis and Functional Probes, College of Biophotonics, School of Optoelectronic Science and Engineering, South China Normal University, Guangzhou 510631, China; MOE Key Laboratory of Laser Life Science & Institute of Laser Life Science, College of Biophotonics, School of Optoelectronic Science and Engineering, South China Normal University, Guangzhou 510631, China; Guangdong Provincial Key Laboratory of Laser Life Science, Guangzhou Key Laboratory of Spectral Analysis and Functional Probes, College of Biophotonics, School of Optoelectronic Science and Engineering, South China Normal University, Guangzhou 510631, China; MOE Key Laboratory of Laser Life Science & Institute of Laser Life Science, College of Biophotonics, School of Optoelectronic Science and Engineering, South China Normal University, Guangzhou 510631, China; Guangdong Provincial Key Laboratory of Laser Life Science, Guangzhou Key Laboratory of Spectral Analysis and Functional Probes, College of Biophotonics, School of Optoelectronic Science and Engineering, South China Normal University, Guangzhou 510631, China; MOE Key Laboratory of Laser Life Science & Institute of Laser Life Science, College of Biophotonics, School of Optoelectronic Science and Engineering, South China Normal University, Guangzhou 510631, China; Guangdong Provincial Key Laboratory of Laser Life Science, Guangzhou Key Laboratory of Spectral Analysis and Functional Probes, College of Biophotonics, School of Optoelectronic Science and Engineering, South China Normal University, Guangzhou 510631, China; MOE Key Laboratory of Laser Life Science & Institute of Laser Life Science, College of Biophotonics, School of Optoelectronic Science and Engineering, South China Normal University, Guangzhou 510631, China; Guangdong Provincial Key Laboratory of Laser Life Science, Guangzhou Key Laboratory of Spectral Analysis and Functional Probes, College of Biophotonics, School of Optoelectronic Science and Engineering, South China Normal University, Guangzhou 510631, China

## Abstract

**Motivation:**

Self-assembling peptides (SAPs), particularly short sequences whose amino acid composition dictates their spontaneous organization, are emerging as versatile building blocks in biomedicine and bio-optoelectronic materials. As the thermodynamic and kinetic behaviours of peptide self-assembly are affected by intrinsic factors, such as amino acid sequence, length, and hydrophobicity, the controllable design of SAPs with tailored properties is essential to achieve specific structures and functions for various application scenarios. However, existing computational approaches, which primarily focus on prediction or heuristic screening, lack conditional generative capability of SAPs.

**Results:**

We present DeepSAP, an end-to-end framework integrating conditional peptide generation with predictive screening for property-guided design of SAPs. By pretraining on large-scale peptide datasets and fine-tuning on limited SAP-specific data, the generative SAPGEN model transfers general sequence knowledge to the SAPs domain, enabling the conditional generation of SAPs in low-data regimes. Subsequently, we constructed a lightweight predictive model, SAPBRF, to further screen the generated peptides. Comparative analyses demonstrated that SAPGEN enabled reliable and precise control of the physicochemical properties of the generated sequence, outperformed baseline generative models in terms of efficiency and quality across multiple design tasks. The SAPBRF predictor achieved efficient and accurate screening with high predictive performance. Finally, the complete DeepSAP framework demonstrated strong generalization under unseen physicochemical regimes, where peptides generated and screened by the pipeline consistently exhibited stable self-assembly behaviour, as further verified by molecular dynamics simulations. Our findings indicate that DeepSAP enables the conditional generation of SAPs with desired properties, holding the potential for tailored design of functional biomaterials.

**Availability and implementation:**

Source code for the DeepSAP model training and evaluation is available at: https://github.com/Paul-hihihopes/Deep_SAP_Design and at DOI: 10.5281/zenodo.21522690.

## 1 Introduction

Self-assembling peptides (SAPs) are promising functional biomaterials owing to their structural diversity, biocompatibility, and precise programmability, enabling diverse applications in drug delivery, regenerative medicine, and bioelectronics (Whitesides and Grzybowski 2002, Gazit 2007, Levin *et al.* 2020, Tian *et al*. 2023). Different biomedical scenarios demand specific thermodynamic and kinetic behaviours of peptide assemblies, which are governed by intrinsic and external factors (Wang *et al.* 2016, Li *et al.* 2021). The intrinsic factors that affect the peptide self-assembly mainly include the sequence of amino acids, number of amino acids, hydrophobicity and charge distribution (Lee et al. 2013a, 2013b, Bortolini *et al.* 2016). Variations in these parameters have been shown to influence the morphology, assembly kinetics, critical aggregation concentration, and environmental responsiveness of self-assembled peptide materials (Bortolini *et al.* 2016, Wang *et al.* 2016, Bao *et al.* 2026, Costa and Zavadlav 2026). However, translating the desired assembly behaviour into concrete sequence choices remains challenging, particularly under complex physiological conditions.

Owing to the scarcity of data, early computational studies on peptide self-assembly relied primarily on molecular dynamics (MD) simulations (Frederix *et al*. 2011, 2015). Although molecular dynamics simulations provide physics-based validation of self-assembly behaviour at the atomic level, their high computational cost makes them better suited as a final validation tool rather than as a routine component of iterative peptide-design workflows. To improve data efficiency, Monte Carlo and active learning strategies have been explored (Shmilovich *et al.* 2020, Batra *et al.* 2022, Xu *et al.* 2023); however, these still depend on expensive simulations or experiments. Given the constraints of simulation-based approaches, subsequent studies sought more efficient screening strategies. Initial approaches leveraged simple physicochemical descriptors, such as net charge and aggregation propensity, as filters to prioritize the candidates (Xiao *et al.* 2021, Kaygisiz *et al.* 2023). Subsequently, the field has evolved to employ machine learning predictors to achieve superior accuracy. A notable advancement in this direction was presented by Njirjak *et al.* (2024), who developed an RNN-based predictor that achieved a higher accuracy in identifying self-assembling peptides. Building upon this predictor, they incorporated it into an evolutionary search framework, enabling guided exploration of the peptide sequence space and successfully generating rational self-assembling peptides. This integrated framework represents an important step towards the data-driven design of SAPs. Nevertheless, evolutionary generators optimize sequences solely through iterative fitness scoring rather than learning the latent-space distribution of peptide sequences. Consequently, they provide constrained diversity and narrow optimization targets, resulting in limited control over the properties of the generated sequence.

With the advent of deep generative modelling, protein design has shifted from passive screening to proactive sequence creation (Keskar *et al*. 2019, Mardikoraem *et al.* 2023). By directly learning the distribution of functional sequences, deep generative models can explore the latent sequence space to produce diverse and reliable candidates with tailored properties (Dathathri *et al.* 2020, Ziegler *et al.* 2023, Wu *et al.* 2024, Koh *et al.* 2025). For example, ProtGPT generates structurally plausible protein sequences through unsupervised learning (Ferruz *et al.* 2022). In addition, reinforcement learning (RL) frameworks have demonstrated the feasibility of controllable protein sequence generation with desired properties (Sternke and Karpiak 2023, Zhang *et al.* 2023). These advances underscore the capability of generative models to capture functional sequence patterns and to design peptides under specific conditions. Despite their success, limitations persist regarding the generation of self-assembled peptides. On one hand, reinforcement learning-based sequence optimization is constrained by its iterative and computationally intensive update loop, rendering it less efficient for large-scale or high-throughput peptide discovery (Wan *et al.* 2024, Wang *et al.* 2024). In contrast, general-purpose protein language models (such as ProtGPT) exhibit problems, such as domain shift and token conflict when transferred to short peptides, resulting in degraded sequence quality (Min *et al.* 2024). Moreover, the extreme scarcity of experimentally validated SAP sequences poses severe challenges for data-driven generative models, undermining their ability to learn meaningful sequence assembly semantics, maintain generation quality, and achieve stable training or regularization.

Transfer learning has emerged as a powerful strategy for addressing small-sample challenges in generative modelling, enabling models pretrained on large datasets to be adapted efficiently to specialized tasks (Rao *et al.* 2019, Georgoulis *et al.* 2025, Yamaguchi *et al.* 2025). However, a dedicated pretrained generative model supporting controllable property-guided generation is still lacking in the peptide domain. Hence, a new peptide-specific generative model, pretrained on large-scale peptide data and subsequently fine-tuned on scarce SAP sequences, offers a promising route towards reliable and controllable self-assembling peptide design.

In this study, we propose DeepSAP, an integrated generative predictive framework for controllable self-assembling peptide design. The principal contributions of this study are threefold: (1) We introduced SAPGEN, a transformer-based generative model pretrained from scratch on a rich peptide corpus. After pretraining, SAPGEN can be efficiently adapted to the SAP domain through lightweight fine-tuning on a very small, curated SAP dataset (342 sequences), enabling a high-quality, property-guided design in settings where data are extremely scarce. (2) We developed a high-throughput predictive module, the SAPBRF, as an accurate and interpretable reward surrogate. Compared with the existing SAP predictor, SAPBRF demonstrates competitive predictive performance on both test and external datasets, making it suitable for large-scale candidate prioritization. (3) We coupled the generator with the predictor as a framework and performed molecular dynamics (MD) validation on peptide candidates filtered under unseen conditions, demonstrating that DeepSAP reliably produces novel SAPs that exhibit physically plausible self-assembly tendencies.

## 2 Methods

### 2.1 Datasets

This study employed two main datasets for training and evaluation: a self-assembling peptide (SAP) dataset and a large-scale bioactive peptide dataset from Peptipedia. Here, we describe the data collection and pre-processing procedures for both datasets.

#### 2.1.1 SAP dataset

For the SAP dataset, positive and negative peptides were collected from three publicly available studies (Xu *et al.* 2023, Njirjak *et al.* 2024, Yang *et al.* 2025). Dataset composition by source is summarized in Table 1. Peptides with terminal modifications and those recorded in nonpolar solvents were excluded to maintain data consistency. To focus on short peptides, where sequence-dependent self-assembly is most prominent, and to ensure sufficient statistical support across length ranges, peptides longer than 15 residues were removed. Duplicates were removed. The final dataset consists of 414 positive and 148 negative peptides, with 20% randomly assigned as an independent test set (83 positive and 29 negative samples). To evaluate model generalization, an external test set was curated (Talluri *et al.* 2025). Pentapeptides annotated as positive in the source study were taken as positive samples, whereas peptides with a β-score below 0.4, as suggested, were regarded as negative samples. The external set included 93 positive and 33 negative pentapeptides after filtering and deduplication, respectively. This external set was used exclusively for independent validation.

**Table 1:** Construction of SAP dataset.

Data source	Original count	Filtered count	Positive samples	Negative samples
**Njirjak *et al.* (2024**)	368	339	219	120
**Yang *et al.* (2025**)	1017	58	36	22
**Xu *et al.* (2023**)	165	165	159	6
**Total**	1529	562	414	148

#### 2.1.2 Peptipedia dataset

For bioactivity-conditioned peptide modelling, sequences were retrieved from Peptipedia v2.0 (Cabas-Mora *et al.* 2024). Only bioactivity types with ≥50 annotated peptides were retained to avoid excessive noise from extremely low-frequency classes. Peptides with ambiguous residues, duplicates, or residues longer than 15 were excluded to ensure consistency with the SAP dataset. To enrich for aggregation-related peptides, amyloid sequences from CPAD 2.0 (Rawat *et al.* 2020) and aggregation-prone peptides from UniProt (UniProt Consortium 2023) were included. After pre-processing, 2 143 263 peptides covering 80 bioactivity types, including antibacterial, anticancer, and anti-inflammatory activities, were split into 80% for training and 20% for validation. Detailed information on the selected peptide types and their quantities is provided in Table 1, available as supplementary data at *Bioinformatics* online.

### 2.2 Overview of DeepSAP

DeepSAP is designed to generate self-assembling peptide candidates under user-defined bioactivity and physicochemical constraints. Given a set of target conditions C, including bioactivity, peptide length, isoelectric point (pI), and GRAVY, the objective is to identify peptide sequences that satisfy the specified physicochemical constraints while exhibiting a high propensity for self-assembly. Formally, the design problem can be expressed as finding a peptide sequence S such that g(S)∈ C, $P_{\emptyset }(Y=1|S)>\gamma$, where g(S) denotes the physicochemical properties of sequence S; Y=1 indicates a self-assembling peptide; $P_{\emptyset }(Y=1|S)$ is the self-assembly propensity predicted by SAPBRF, and γ denotes the predefined decision threshold.

The DeepSAP framework addresses this design problem through a sequential computational workflow (Fig. 1). First, SAPGEN generates candidate peptide sequences conditioned on the specified bioactivity and physicochemical constraints according to the conditional distribution. The generated candidates are then evaluated by SAPBRF to estimate their self-assembly propensity and to identify high-scoring candidates. At the sequence-generation level, SAPGEN models the conditional probability of a peptide sequence autoregressively as:


(1) $$P_{\theta }(S|C)=\prod_{i=1}^{L}P_{\theta }(s_{i}|s_{<i},C),$$

where $S=s_{1}\ldots s_{L}$ denotes the generated peptide sequence, $s_{i}$ represents the preceding amino acid tokens, L is the sequence length, and C represents the conditioning information.

**Figure 1:**
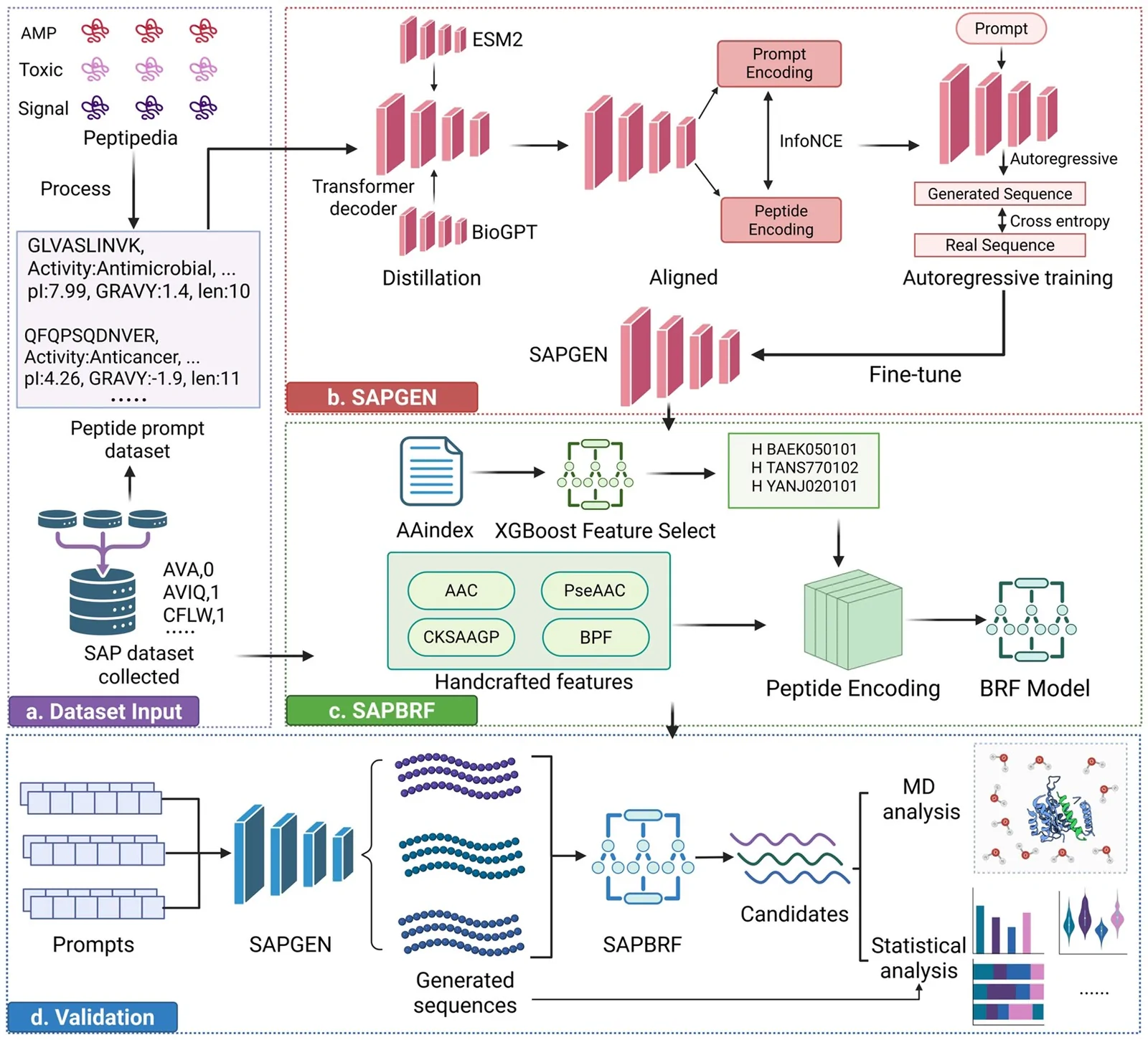
Overall workflow of the proposed DeepSAP framework. (a) Construction of peptide datasets: peptides were collected from curated SAP studies and the Peptipedia database. Physicochemical properties of peptides were calculated and encoded into prompts. (b) Model training: the model was trained in three phases: distillation (teacher models: BioGPT (Luo *et al.* 2022) for prompts, ESM2 (Lin *et al.* 2023) for sequences representation), contrastive learning using the InfoNCE loss (van den Oord *et al*. 2018), and autoregressive training, followed by fine-tuning on the SAP dataset. (c) Construction of the predictor: peptide encoding comprised four handcrafted characteristics: amino acid composition (AAC), pseudo amino acid composition (PAAC), composition of k-spaced amino acid group pairs (CKSAAGP), and binary profile features (BPF). Feature importance analysis was performed using XGBoost (Chen and Guestrin 2016). On the physicochemical indices from AAIndex database (Kawashima and Kanehisa 2000) and construct feature vectors. A Balanced Random Forest (BRF) model was used as the predictor (Chen *et al.* 2004). (d) Model validation process through statistical and MD analyses.

SAPGEN encodes bioactivity and physicochemical conditions as structured prompts using dedicated special tokens in a unified vocabulary. Each peptide was associated with a structured prompt consisting of bioactivity tokens drawn from the 80 categories described in Section 2.1, together with physicochemical property tokens, including isoelectric point [pI, predicted by IPC2.0 (Kozlowski 2021)], hydrophobicity [GRAVY, (Kyte and Doolittle 1982)], and sequence length. Both pI and GRAVY were Discretized into equal-width intervals of 0.5, based on their empirical ranges in the training corpus. The sequence length was encoded as a discrete token from 2 to 15. Values outside the predefined ranges were clipped and mapped to the nearest boundary bin. This design allows conditioning tokens to directly influence sequence generation through causal self-attention across all the decoding steps. To preserve the minimal unit information, the model tokenized peptide sequences into single amino acid tokens based on 20 standard amino acids. Formally, each peptide is represented as:


(2) $$\mathrm{Peptide}=f_{1}+f_{2}+...+f_{k}+\mathrm{pI}+L+G:\;S,$$

where fi denotes the ith peptide activity descriptor, S represents the peptide sequence consisting of amino acid tokens, G represents the computed GRAVY values, and L represents the peptide length.

### 2.3 SAP model architecture

To model the conditional generation of short peptides under specific bioactivity and physicochemical constraints, we developed SAPGEN, a transformer-based generative model tailored for conditional peptide generation. The core architecture of SAPGEN consists of a customized transformer decoder comprising 12 transformer blocks, each with a hidden size of 512, and 16 attention heads. Peptide sequences were generated in an autoregressive manner using a conditional prompt. The architecture and hyperparameters of the model in detail are listed in Table 3, available as supplementary data at *Bioinformatics* online.

### 2.4 SAPGEN pretraining strategy

Given the scarcity of self-assembling peptide (SAP) data, SAPGEN was first pretrained on a large-scale general peptide dataset (Section 2.1) to acquire general peptide sequence patterns and conditional generation capabilities. In the pretraining process, we introduced a three-stage training strategy to enhance both generation fidelity and controllability. The pretraining process included knowledge distillation, contrastive learning, and autoregressive training. During these stages, SAPs sequences were included in the distillation and contrastive phases to enhance representation quality but were excluded from autoregressive training to avoid over memorization.

The initial semantic representations of peptides and prompts affect the subsequent training process (Bengio *et al.* 2013). Knowledge distillation enables the student model to learn pretrained prior information from the teacher models (Hinton *et al*. 2015). To capture meaningful representations of peptide sequences and structured prompts, BioGPT (Luo *et al.* 2022) and ESM2-150M (Lin *et al.* 2023) were employed as teacher models. BioGPT is a sophisticated language model that is pretrained on the rich biomedical literature. ESM2-150M is a protein language model that is pretrained on a large corpus of protein sequences. Distillation was performed using the Peptipedia training set (Section 2.1). During distillation, the student model encodes the prompt and sequence representations that are projected through a linear layer to match the dimensions of the teacher outputs. We then used the mean squared error (MSE) calculated between the student and teacher hidden representations as a loss function to align the feature space of the students with that of teachers. Distillation loss is defined as follows:


(3) $$L^{KD}=\alpha \cdot \mathrm{MSE}(h_{G},h_{Sp})+\beta \cdot \mathrm{MSE}(h_{E},h_{Ss}),$$

where $h_{G}$ and $h_{E}$ denote the hidden representations produced by BioGPT and ESM2, respectively, and $h_{\mathrm{Sp}}$ and $h_{\mathrm{Ss}}$ represent the corresponding prompt and peptide sequence representations generated by the student model.

After distillation, the embeddings of the prompts and peptides were aligned with the semantic spaces of BioGPT and ESM2, respectively. In the contrastive learning phase, we further aligned these two modality-specific representations and projected them onto a unified embedding space (van den Oord *et al*. 2018). We used CLIP (van den Oord *et al*. 2018), a classical contrastive learning framework, to construct an association between the prompts and peptides. In this phase, the model was optimized to pull together representations of matched prompt-peptide pairs while pushing apart mismatched pairs within each training batch. The model first encodes sequence-prompt pairs in batches and then uses symmetric InfoNCE loss (van den Oord *et al*. 2018) to facilitate bidirectional alignment between the prompt and peptide embeddings:


(4) $$L^{I}=-\log\frac{\exp\left(\frac{\mathrm{sim}(z_{i},z_{i}^{+})}{\gamma }\right)}{\sum_{j=1}^{N}\exp\left(\frac{\mathrm{sim}(z_{i},z_{j})}{\gamma }\right)}.$$

Here, $z_{i}$ and $z_{i}^{+}$ represent the latent embeddings of the prompt and its corresponding sequence produced by the model, sim() denotes cosine similarity, γ is the temperature hyperparameter, and N is the number of samples in a batch. The contrastive objective is defined symmetrically over both the prompt-to-sequence and sequence-to-prompt directions, as follows:


(5) $$L^{CL}=\frac{1}{2}(L^{p\to s}+L^{s\to p}),\;\;$$

where $L^{p\to s}$ and $L^{s\to p}$ denote the InfoNCE losses computed by treating prompts and sequences as queries respectively.

During the previous two stages, the model learns an aligned semantic representation. We then trained SAPGEN to integrate the representations into a conditional generative framework. In the autoregressive training phase, the model learns to generate peptide sequences token-by-token, conditioned on structured prompts. The training objective is the cross-entropy loss between the predicted token distribution and the ground truth sequence tokens, which can be formulated as follows:


(6) $$L^{CE}=-\frac{1}{N}\sum_{i}^{N}\left[y_{i}\cdot \log\left(q_{i}\right)+(1-y_{i})\cdot \log\left(1-q_{i}\right)\right],$$

where yi represents the label of the ith amino acid, equivalent to the token value of the input sequence, and qi represents the output probability distribution of the amino acid at position.

The knowledge distillation phase was conducted for 5 epochs with a batch size of 1024, we set α = 0.5 and β = 1.0 to balance the contributions of prompt and sequence level supervision. The subsequent contrastive alignment phase was trained for 20 epochs with a temperature parameter γ = 0.03 and a batch size of 512. The final autoregressive training phase employed a batch size of 1024. All training stages used the AdamW optimizer (Loshchilov and Hutter 2019) with an initial learning rate of 1e − 4, cosine annealing learning rate scheduling (Loshchilov and Hutter 2017), and linear warm-up over the first 5% of the training steps. All the experiments were conducted on an NVIDIA RTX 4090 GPU, and the complete training process required ∼ 14 h. All scripts are publicly available at https://github.com/Paul-hihihopes/Deep_SAP_Design.

### 2.5 Fine-tuned process of SAPGEN

The pretrained model was fine-tuned to transfer its conditional peptide generation capability to SAP generation. Specifically, we constructed a fine-tuning dataset by combining 75% of the original pretraining peptide sequences with 25% of the experimentally validated self-assembling sequences from the SAPBRF training set to mitigate overfitting because of the limited number of positive examples. We retained the same structured prompt format used in pretraining, with the bioactivity token fixed to ‘Self assembly’ while preserving the physicochemical property tokens (e.g. GRAVY score and peptide length) to allow conditional generation. Fine-tuning was performed using the AdamW optimizer with a learning rate of 2 × 10^−^^5^, a batch size of 8, and 20 epochs.

### 2.6 SAPBRF model

Although ML-SAP offers a sequence-based approach for aggregation prediction, its susceptibility to class imbalance may restrict its applicability in large-scale generative screening. To evaluate the self-assembly potential of a large number of generated peptides efficiently and accurately, we developed the SAPBRF. To characterize peptide sequences for self-assembly prediction, we constructed a unified feature representation of peptides by integrating sequence-derived, physicochemical, and model-based descriptors. First, four widely used handcrafted features were computed, including amino acid composition (Chou 2001), k-spaced group-pair composition (Chen *et al.* 2007), pseudo amino acid composition (Chou 2001), and binary profile encoding (Shen and Chou 2008), collectively capturing global residue composition and local sequence-order patterns. Physicochemical properties were extracted from the AAindex database (v9.2, (Kawashima and Kanehisa 2000)). The top ten most informative indices were selected using XGBoost (Chen and Guestrin 2016) feature importance and aggregated across residues to form a compact physicochemical representation (the full index list is provided in Table 2, available as supplementary data at *Bioinformatics* online). The concatenation of handcrafted descriptors and AAindex-derived properties resulted in a 438-dimensional feature vector for each peptide, which served as the input to SAPBRF classifier (see Supplementary S1 for full details on feature derivation and processing).

**Table 2:** SAP design tasks.

Task name	Function Activity	pI bin	Len	GRAVY bin	num samples
**Sa1**	Self assembly	5.5–6.0	4	2.5–3.0	500
**Sa2**	Self assembly	5.5–6.0	4	0.5–1.0	375
**Sa3**	Self assembly	3.5–4.0	6	1.0–1.5	400
**Sa4**	Self assembly	3.5–4.0	6	1.5–2.0	225
**Sa5**	Self assembly	8.0–8.5	15	1.0–1.5	125
**Sa6**	Self assembly	8.0–8.5	10	0.0–0.5	100
**Sa7**	Self assembly	6.0–6.5	5	0.0–0.5	75
**Sa8**	Self assembly	6.0–6.5	7	−3.0–-2.5	75
**Sa9**	Self assembly	7.0–7.5	4	1.5–2.0	100
**Sa10**	Self assembly	7.0–7.5	6	1.0–1.5	30

**Table 3:** Model performance verification.

	Baseline	BRF	XGB	LGB	ProtBERT-LoRA	ESM2-LoRA
**ACC**	0.8388 ± 0.023	**0.901 ± 0.0143**	0.892 ± 0.0217	0.879 ± 0.0116	0.7943 ± 0.0389	0.8774 ± 0.0098
**SN**	0.9 ± 0.011	0.904 ± 0.0251	**0.948 ± 0.0217**	0.942 ± 0.0209	0.7943 ± 0.0389	0.8774 ± 0.0098
**SP**	0.75 ± 0.023	**0.906 ± 0.0134**	0.744 ± 0.0541	0.710 ± 0.0447	0.5793 ± 0.3124	0.8069 ± 0.0634
**AUC**	0.8889 ± 0.016	**0.9729 ± 0.01**	0.951 ± 0.0110	0.947 ± 0.0121	0.8418 ± 0.0184	0.915 ± 0.0092
**F1**	0.9 ± 0.012	**0.930 ± 0.0111**	0.927 ± 0.0150	0.916 ± 0.0087	0.7738 ± 0.0876	0.8783 ± 0.0078

Note: Bold values indicate the best performance for each evaluation metric.

### 2.7 Evaluation

#### 2.7.1 Conditional generation evaluation settings

SAPGEN was evaluated under two conditional generation settings. In the single-attribute setting, the model is conditioned on the self-assembly objective together with one target physicochemical attribute, namely peptide length, pI, or GRAVY. In the joint-attribute setting, the model is conditioned simultaneously on the self-assembly objective and simultaneous constraints on all three attributes.

#### 2.7.2 Hit rates

For each individual attribute—peptide length (L), pI (P), and GRAVY (H), the hit rate of attribute is defined as the proportion of generated sequences satisfying the corresponding target constraint. The joint hit rate is defined as the proportion of generated sequences that simultaneously satisfy all specified physicochemical constraints. For pI and GRAVY, a tolerance of ±0.1 is applied when determining constraint satisfaction to account for prediction uncertainty.

In addition to attribute-based hit rates, predictor-based hit rates were used to assess the self-assembly propensity of generated peptides. The SAPBRF and ML-SAP (Njirjak *et al*. 2024) hit rates were defined as the proportions of generated sequences classified as self-assembling by the respective predictors. A decision threshold of >0.5 was used for SAPBRF, whereas a threshold of >0.9 was used for ML-SAP (Supplementary S4).

#### 2.7.3 Performance evaluation metrics and statistical analysis

Besides attribute-based hit rate and predictor-based hit rate, generated peptides were evaluated using BLOSUM62 similarity, normalized Levenshtein distance, and sequence uniqueness (Levenshtein 1966, Henikoff and Henikoff 1992). The SAP predictor was assessed using accuracy (ACC), sensitivity (SN), specificity (SP), F1 score (Powers 2011), and area under the receiver operating characteristic curve (AUC) (Hanley and McNeil 1982). Detailed definitions and calculation procedures are provided in Supplementary S10.

Quantitative results were obtained from independent computational runs. For generative model evaluation, including attribute controllability testing, baseline comparisons, and ablation analyses, the corresponding evaluation metrics were calculated from five independent runs. For predictive model evaluation, performance metrics were calculated from ten independent runs using different random seeds. Where multiple independent runs were performed, evaluation metrics were calculated independently for each run and summarized as mean ± SD. Comparisons of generative/predictive models with its baselines, and ablation analyses were assessed using the two-sided Wilcoxon signed-rank test (*P *< .05).

#### 2.7.4 Molecular dynamics-based aggregation evaluation

The self-assembly behaviour of generated peptides was evaluated using coarse-grained molecular dynamics (CGMD) simulations based on the Martini 2.2P force field. Aggregation propensity (AP) was used to quantitatively assess peptide aggregation (Frederix *et al.* 2011). An AP value of ≥1.75 was adopted as the predefined criterion for aggregation, consistent with previous work (Njirjak *et al.* 2024). Final-frame morphologies were also examined as complementary structural evidence of peptide aggregation. The detailed definition and calculation of AP, together with the CGMD simulation protocols and parameters, are provided in Supplementary S5.

## 3 Results

### 3.1 SAPGEN enables the control of physicochemical properties of the generated peptide

To evaluate the controllability of SAPGEN in generating peptides with the targeted properties, we evaluated the model under predefined single-attribute and joint-attribute generation settings (Section 2.7.1), with the target values or ranges for each physicochemical property shown in Fig. 2a.

**Figure 2:**
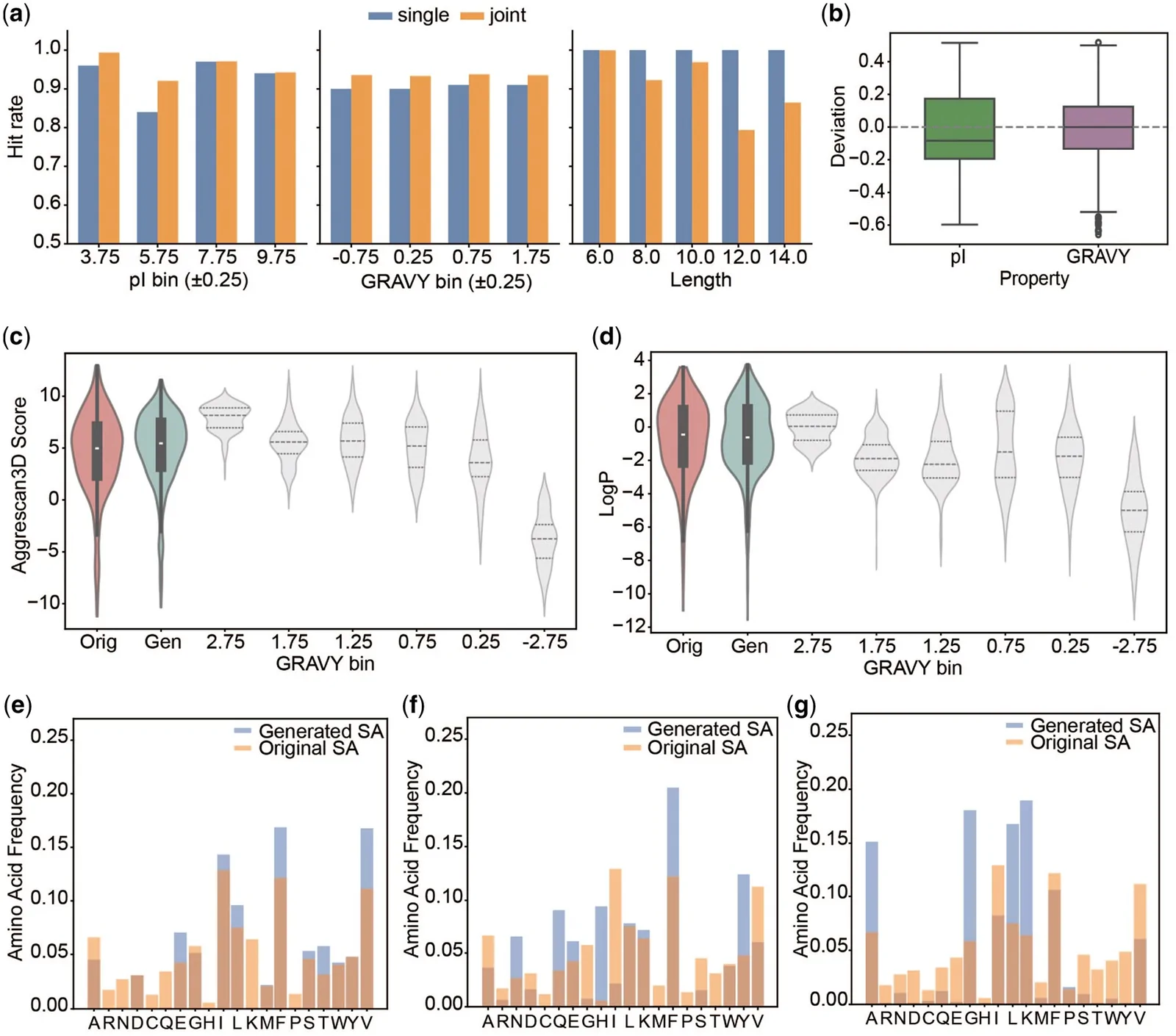
Controllability of SAPGEN in conditional peptide generation. (a) Hit rate performance of the model in single-attribute and joint-attribute generation tests across various property targets. (b) Distribution of deviations of property values for joint-attribute conditioned peptide generation. (c, d) Comparison of physicochemical property distributions (Aggrescan3D score, LogP) between the original sequences and generated sequences, across different target GRAVY bins. (e, f) Amino acid compositions of self-assembled peptides generated by the model under different pI conditions (acidic, intermediate, and basic), compared with the original self-assembling peptide sequences.

As shown in Fig. 2a, SAPGEN exhibits excellent controllability in a single-attribute setting, achieving a perfect hit rate across all targeted lengths and high accuracy for physicochemical properties, with mean hit rates of 0.928 for pI and 0.905 for GRAVY across all evaluated bins (*n* = 5). Under joint constraints on multiple attributes, SAPGEN maintained strong overall controllability, with a joint hit rate of 0.811 across all properties. Notably, the pI and GRAVY compliance remained robust in the joint setting (mean hit rates of 0.957 and 0.935, respectively). Length accuracy experienced reduction under joint constraints (−8.8% relative), with the decline most evident for longer peptides (12–14 residues), yet still exceeding 0.78 in affected bins. Figure 2b shows the distribution of the prediction deviations for pI and GRAVY in the joint attribute generation. The deviations were calculated as the difference between the predicted values of the generated peptides and midpoints of their target intervals. For both properties, most of the generated peptides had relatively small deviations (<0.2). The average error for pI was −0.031, while that for GRAVY was −0.005, suggesting that the model faced more difficulty in precisely controlling the pI of the generated peptides. Overall, the results demonstrate that SAPGEN preserves high property controllability in both single- and joint-attribute generation, highlighting its capability to support the flexible and precise physicochemical design of self-assembling peptides.

To further assess the ability of the model to generate rational peptides under tailored physicochemical conditions, we selected several classical scenarios as targets and observed their performance. First, we examined the distribution of SAP properties in the original dataset (Table 5, available as supplementary data at *Bioinformatics* online). Based on the distribution of SAP properties in the original dataset, we selected ten representative prompts for generation, covering a wide range of pI values, peptide lengths, and hydrophobicity levels (Table 2). High-frequency prompts, such as pI 5.5–6.0 with short peptides and moderate GRAVY (Sa2), were assigned larger generation quotas to match the dataset distribution, while medium- and low-frequency prompts were included to test the performance of the model under less common conditions, including long basic peptides (Sa5), strongly hydrophilic sequences (Sa8), and rare prompts with minimal representation (Sa10).

We analysed the physicochemical properties of the generated peptides (Fig. 2c and d). We used LogP (Hansch *et al.* 1995) and Aggrescan3D (A3D) scores to assess the overall hydrophobicity and aggregation propensity, respectively. The median A3D and LogP values generally exhibited monotonic trends across the GRAVY categories; peptides generated under higher GRAVY constraints tended to have higher A3D scores and more positive LogP values. Moreover, the overall A3D and LogP distributions of the generated sequences closely mirrored those of the original dataset, suggesting that SAPGEN successfully preserved the overall distribution of physicochemical properties while introducing controlled hydrophobic variations.

We further examined the amino acid composition of the generated peptides across three pI ranges—acidic (pI = 3.5–5.5), neutral (pI = 5.5–7.5), and basic (pI = 7.5–9.5)—to assess the ability of the model to perform conditionally controlled sequence generation (Fig. 2e–g). In the acidic range, the generated sequences were enriched in negatively charged residues, whereas the basic range exhibited higher proportions of positively charged residues, and the neutral range displayed a more balanced mix of polar and hydrophobic residues. The higher frequency of small neutral residues, such as Ala and Gly, in the basic range is consistent with the amino acid distribution observed in the original training dataset. Notably, the occurrence frequencies of key hydrophobic residues, including phenylalanine (F) and leucine (L), remained at a reasonable level across all pI ranges, suggesting that the model reallocates amino acid composition in response to pI constraints, while preserving hydrophobic features relevant to peptide self-assembly.

### 3.2 SAPBRF enables accurate and efficient screening of self-assembling peptides

To ensure that evaluation of the generated peptides relied on a robust and reliable computational criterion, we assessed the predictive performance of the SAPBRF model employed through this study. As shown in Table 3, on a rigorously curated independent internal test set, SAPBRF achieved an accuracy of 0.9019 ± 0.0143 and an AUC of 0.9729 ± 0.01 (Fig. 3b), outperforming the baseline, other machine learning methods [XGB, LGB (Ke *et al.* 2017)], as well as fine-tuned protein language models (ProtBERT-LoRA (Elnaggar *et al.* 2021, Hu *et al.* 2021) and ESM2-LoRA, Supplementary S8) across all metrics except sensitivity (*P *< .05). Notably, SAPBRF showed markedly improved handling of class imbalance, as evidenced by substantial gains in minority-class recall compared with the baseline. Moreover, in external validation, the model yielded an accuracy of 0.9543 ± 0.0037 and an AUC of 0.9792 ± 0.0164, both surpassing the baseline ML-SAP predictor (Fig. 3c). Together, these results suggest that SAPBRF provides a highly reliable and discriminative assessment of the peptide self-assembly propensity, serving as a solid foundation for downstream evaluation. In terms of inference efficiency, SAPBRF completed inference on 1000 peptides in 7.07 ± 1.15 s using an AMD Ryzen 95 950X CPU, indicating efficient computational performance. For details regarding model interpretability and counterfactual analysis (Wachter *et al.* 2017), please refer to Supplementary S2 and S3.

**Figure 3:**
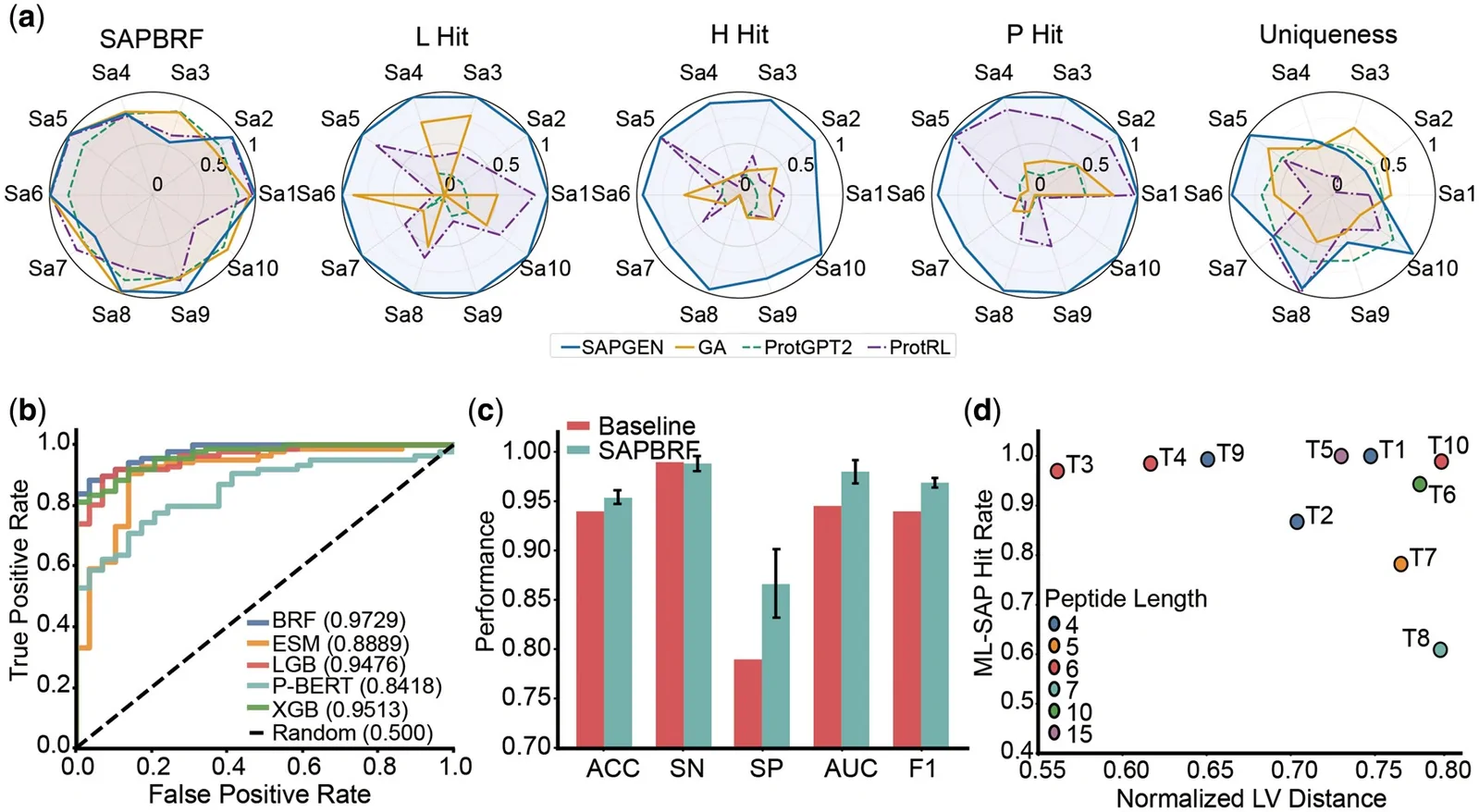
Performance evaluation of SAPGEN and SAPBRF. (a) Radar plots comparing the performance of the proposed generative model and baseline GA model across five metrics—SAPBRF hit rate, L Hit (length constraint hit rate), H Hit (GRAVY constraint hit rate), P Hit (pI constraint hit rate), and redundancy—for ten generation tasks (*n* = 5). (b) ROC curves of different predictive models evaluated in independent test set (*n* = 10). (c) Bar plots comparing the external test performance of SAPBRF and the baseline model across ACC, SN, SP, AUC, and F1 metrics. (d) ML-SAP hit rates and normalized Levenshtein distance of the generative model for ten generation tasks.

### 3.3 Performance of SAPGEN under conditional peptide generation tasks

To evaluate the performance of fine-tuned SAPGEN in conditional peptide generation, we comprehensively compared it with three baseline models (Supplementary S7) across the ten conditional generation tasks (Sa1–Sa10), as shown in Table 4 and Fig. 3a. SAPGEN demonstrated substantially superior controllability across all three conditional constraints. In contrast, all three baseline models exhibited markedly lower hit rates across these constraints (Fig. 3a, *P *< .05). ProtRL (Stocco *et al.* 2025) showed stronger conditional control than GA and ProtGPT2 (Ferruz *et al.* 2022), suggesting that reinforcement learning can facilitate conditional peptide generation. However, its performance remained substantially below that of SAPGEN across all three constraints, suggesting that multi-objective optimization based on RL still presents challenges for SAP generation. Importantly, the enhanced controllability of SAPGEN was accompanied by favourable overall generation performances. SAPGEN achieved a mean SAPBRF hit rate compare to that of GA, but higher than those of ProtGPT2 and ProtRL. This indicates that the improvement in constraint satisfaction did not occur at the expense of overall sequence quality. Notably, SAPGEN also achieved the highest sequence uniqueness among the four models (Fig. 3a). This highlighted the effectiveness of SAPGEN for sequence generation.

**Table 4:** Quantitative comparison of SAPGEN and baseline models.

Model	SAPBRF Hit	L Hit	H Hit	P Hit	Uniqueness
**SAPGEN**	**0.8778 ± 0.1617**	**0.9995 ± 0.0017**	**0.9071 ± 0.0735**	**0.9737 ± 0.0485**	**0.6897 ± 0.2558**
**Baseline GA**	**0.8939 ± 0.0898**	0.4240 ± 0.3426	0.2710 ± 0.1554	0.2580 ± 0.2379	0.5251 ± 0.1435
**ProtGPT2**	0.8350 ± 0.0193	0.1619 ± 0.0978	0.1703 ± 0.0585	0.2176 ± 0.1637	0.6042 ± 0.0971
**ProtRL**	0.8342 ± 0.1659	0.5151 ± 0.2399	0.3333 ± 0.2637	0.5903 ± 0.3505	0.4266 ± 0.2970

Note: Bold values indicate the best or second-best performance for each evaluation metric.

**Table 5:** Ablation analysis.

Model	SAPBRF	ML-SAP	BLOSUM62	LV Dist	Uniqueness
**Full model**	**0.8777 ± 0.138**	**0.845 ± 0.164**	**2.83 ± 0.38**	**4.22 ± 2.07**	**0.7121 ± 0.236**
**Model without distillation**	0.7398 ± 0.131	0.6124 ± 0.310	2.71 ± 0.34	3.33 ± 1.13	0.6409 ± 0.287
**Model distillation with ESM2 only**	0.7974 **±** 0.074	0.7630 **±** 0.266	2.78 **±** 0.22	4.17 **±** 1.27	0.6983 **±** 0.225
**Model without alignment**	0.8108 ± 0.130	0.7429 ± 0.126	2.76 ± 0.31	4.15 ± 1.39	0.5505 ± 0.279

Note: Bold values indicate the best performance for each evaluation metric.

Figure 3d shows the performance of the fine-tuned SAPGEN on ten conditional generation tasks evaluated using the ML-SAP predictor. SAPGEN achieved a high average ML-SAP hit rate (0.845 ± 0.027). Simultaneously, the model maintained substantial sequence diversity with a normalized Levenshtein distance of 0.7515. Moreover, in terms of inference efficiency, as the GA optimization process is no longer required, average time to generate one peptide using SAPGEN is only 0.032 ± 0.0183 s, while the baseline model takes 4.397 ± 0.414 s, achieving a speedup of two orders of magnitude. Overall, SAPGEN demonstrated reliable and efficient conditional peptide generation with superior controllability, diversity, and inference efficiency under the observed conditions.

### 3.4 Performance of DeepSAP pipeline in multiple physicochemical regimes

To evaluate the applicability and generalization of our DeepSAP pipeline, we performed two targeted case studies on the design of self-assembled peptides under unseen physicochemical constraints. In this study, we aimed to generate longer peptides with near-neutral properties, using two prompts: (1) Self-assembly, Length: 10, pI: 7.0–7.5, and GRAVY: −0.5–0.0; and (2) Self-assembly, Length: 8, pI: 7.0–7.5, and GRAVY: −0.5–0.0. These tasks target weakly basic peptides with moderate hydrophobicity, potentially allowing the modulation of the self-assembly behaviour through pH-sensitive interactions. For each prompt, we generated 2000 candidate sequences using the SAPGEN model. The resulting sequences were subsequently filtered using the proposed SAPBRF predictive model. For each generation task, we selected the five sequences with the highest predictive scores for further validation.

Across both novel generation tasks, SAPGEN achieved high fidelity to the target GRAVY (−0.5 to 0.0) and pI constraints, with GRAVY hit rates of 86.6% and 93.0% and pI hit rates of 90.5% and 82.6% for Tasks 1 and 2, respectively (Fig. 4a). Then, all ten selected peptides were subjected to MD simulations to examine their self-assembly behaviour. As shown in Fig. 4b and c, all tested peptides exhibited AP values greater than 2.0, confirming their strong self-assembly tendency, whereas all simulation results showed compact, ordered aggregates. The predicted scores showed a weak association with aggregation propensity, and a strict linear relationship was not observed. Detailed validation results of peptides generated from the two unseen prompts are provided in Supplementary Table 6, available as supplementary data at *Bioinformatics* online. Together, these results demonstrate that our DeepSAP framework can reliably obtain peptides that satisfy the specified physicochemical constraints and exhibit consistent self-assembly tendencies at the molecular level.

**Figure 4:**
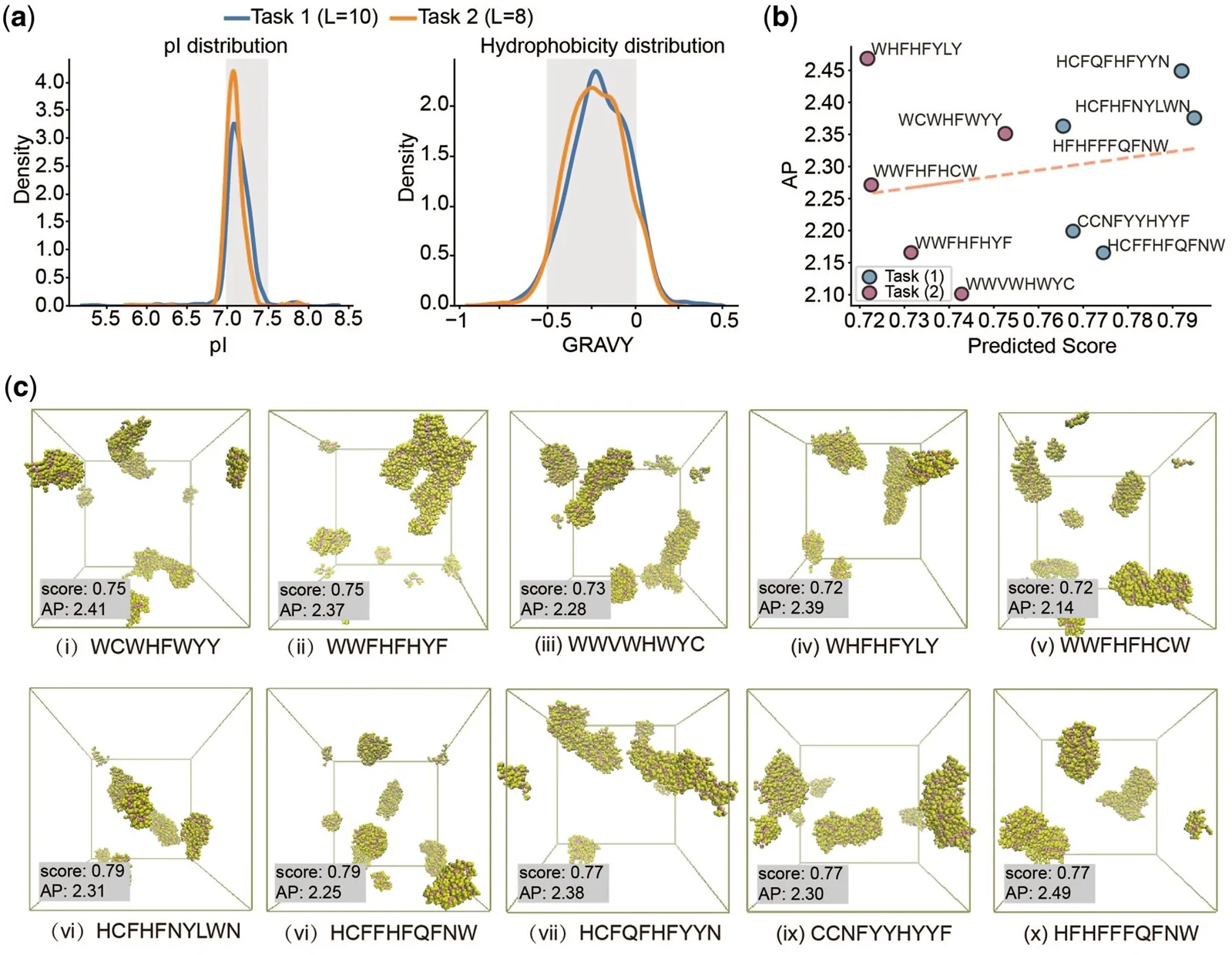
Validation of the DeepSAP pipeline under previously unseen physicochemical constraints. (a) Distributions of the isoelectric point (pI) and GRAVY for 2000 peptides generated for two previously unseen generation tasks. (b) Distribution of APSASA values and SAPBRF predicted scores of screened peptides. (c) Final conformations of the screened peptides evaluated by molecular dynamics simulations, together with the corresponding SAPBRF predicted scores and APSASA values.

To further evaluate the robustness of the proposed pipeline, we also performed CGMD simulations for peptides generated from all ten representative SAP design tasks (Sa1–Sa10). Among the 30 peptides evaluated, 27 satisfied the aggregation criterion (AP ≥ 1.75), demonstrating that the proposed pipeline can reliably identify self-assembling peptide candidates across diverse conditional generation tasks. Detailed simulation results are provided in Supplementary S9.

### 3.5 Robustness and ablation analysis of SAPGEN under physicochemical constraints

To evaluate the robustness of our DeepSAP framework under various physicochemical conditions, we generated peptides across a combinatorial matrix of four chain lengths (4, 6, 8, and 10 residues), four target pI intervals (3.5–4.0, 5.0–5.5, 7.0–7.5, and 9.0–9.5) and four GRAVY intervals (−1.5–−1.0, −0.5–0.0, 0.0–0.5, and 1.0–1.5). For each case, 1000 sequences were generated for downstream analyses.

We first observed the peptide generation performance of SAPGEN under various conditions, as evaluated using SAPBRF (Fig. 5a). Across all four length constraints, a consistent low-scoring pattern emerged in the upper-left region of the heat map. Specifically, peptides with low pI (3.5–4.0) combined with low GRAVY (−1.5–0.0), together with those at pI 5.0–5.5 and highly hydrophilic GRAVY (−1.5 to −1.0), form a characteristic L-shaped low hit rate region. Beyond these local effects, a global trend is observed along the pI and GRAVY axes. Peptides generated at higher pI intervals and higher GRAVY values consistently achieve higher SAPBRF logits across length constraints, indicating that elevated net charge and increased hydrophobicity jointly favour the predicted self-assembly propensity. Although peptides of most lengths consistently achieved hit rates above 0.8 under all tested property combinations, the generated hexapeptides performed poorly under all tested property combinations. To further validate the generalizability of the generated sequences, we evaluated them using an independent predictor, ML-SAP, which yielded an overall hit rate of 0.7106 ± 0.2628 across all conditions. This indicates that direct generation of peptides with robust self-assembly capabilities remains relatively difficult in various complex scenarios. Subsequently, we employed SAPBRF as a post-generation filtering module to rank the candidates and retain the top 20 sequences for each physicochemical condition. This screening strategy significantly improved the average hit rate on the independent ML-SAP predictor to 0.9422 ± 0.17 (Supplementary S5), which demonstrates that the inclusion of the predictor can improve the robustness of the overall generation process. In summary, these results demonstrate that the DeepSAP pipeline can reliably generate self-assembled peptides of diverse lengths and physicochemical properties.

**Figure 5:**
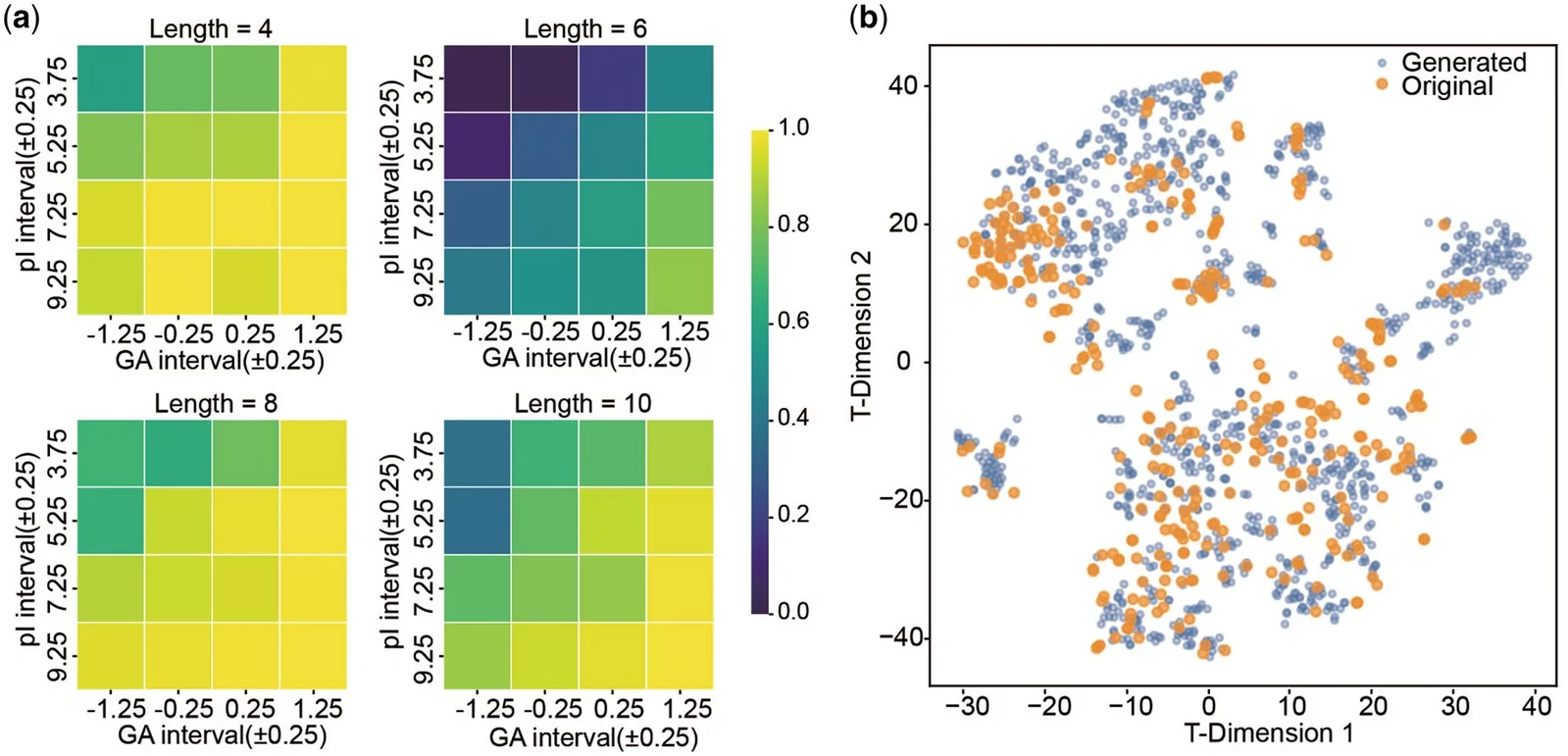
Robustness and t-SNE analysis of SAPGEN under diverse physicochemical constraints. (a) Heat map of SAP hit rates for generated self-assembled peptides conditioning on different length, pI and GRAVY constraints, evaluated by SAPBRF. (b) t-SNE visualization of ESM2 embedding of SAPGEN generated peptides and original self-assembling peptides in ten generation tasks.

To explore the contributions of each pretraining stage, we conducted ablation experiments by progressively removing the knowledge distillation and alignment components from the full model. The comparative experiments were performed on the same ten conditional generation tasks (Sa1–Sa10) as detailed in Table 2. The results are summarized in Table 5. The full training model achieved the best overall performance, achieving high hit rate on both predictors (SAPBRF: 0.8777, ML-SAP: 0.85, *n* = 5). Removing the knowledge distillation objective led to the most significant performance degradation across almost all metrics. SAPBRF decreased by approximately 18.5% and ML-SAP decreased by approximately 27.5%. This indicates that knowledge distillation plays a crucial role in stabilizing training and improving the fidelity and semantic consistency of generated sequences. Then again, removing BioGPT and using only ESM2 for knowledge distillation, which also resulted in a degraded performance, with SAPBRF decreasing from 0.8777 (dual-teacher) to 0.7974 ± 0.074 (ESM2-only) and ML-SAP from 0.8450 to 0.7630, suggesting the benefit of integrating prompt-level and sequence-level supervision during distillation. In contrast, removing the alignment objective resulted in a relatively smaller performance degradation. SAPBRF and ML-SAP hit rates decreased by approximately 10.7% and 12.1% (*P* < .05), respectively, while the uniqueness decreased significantly (from 0.7121 to 0.5505), indicating that the alignment training helps increase diversity in sequence generation. Overall, the ablation results demonstrate that both knowledge distillation and alignment objectives are necessary for optimal performance, with their combination yielding the most stable and high-quality peptide generation in the downstream tasks.

To further examine whether SAPGEN preserves the underlying semantic space of self-assembling peptides, we projected both generated sequences in ten tasks and original validated sequences into a shared embedding space using ESM2 representations, followed by t-SNE visualization [Fig. 5b (van der Maaten and Hinton 2008)]. The resulting distribution shows that the SAPGEN-generated peptides substantially overlap with the original SAPs, indicating that the model captures sequence semantics similar to those of the self-assembled peptides. Meanwhile, the generated sequences extended into adjacent regions of the latent space without forming isolated clusters, suggesting controlled exploration beyond the training distribution rather than trivial memorization. Together, these results support the fidelity and diversity of peptides generated by SAPGEN under physicochemical constraints.

## 4 Discussion

### 4.1 Physicochemical interpretation of self-assembly revealed by SAPBRF

Our predictive module (SAPBRF) provided interpretable physicochemical insights into the mechanisms of peptide self-assembly. Feature importance analysis indicated that chain flexibility, helical structure propensity, side-chain conformation, and local hydrophobicity (e.g. H BAEK050101, H TANS770102, H YANJ020101, and H PONP800103) were the dominant determinants of self-assembly, consistent with established mechanisms in which β-sheet stabilization and hydrophobic packing drive peptide aggregation. Conversely, proline (P)—an intrinsic breaker for β sheet formation—was consistently associated with reduced assembly propensity, agreeing with its known structural-disruptive role. Taken together, these observations indicate that the predictive model captures meaningful biophysical rules, thereby supporting its use as a reliable model for evaluating large-scale generative outputs.

Interestingly, all machine learning models based on handcrafted features—including SAPBRF, XGBoost, and LightGBM—consistently outperformed fine-tuned protein language models. This suggests that the prediction of peptide self-assembly relies primarily on amino acid composition and overall physicochemical properties rather than on sequence-based contextual information. To test this hypothesis, we conducted a sequence-shuffling experiment in which the amino acid order was randomized while the composition remained unchanged. The results showed that SAPBRF still achieved a high AUC of 0.9511, compared to 0.9729 on the original data; this finding aligns with recent research by Tamás *et al.* (2026), which demonstrated that amino acid composition alone is sufficient to predict peptide aggregation behaviour during solid-phase synthesis. Our study extends this conclusion to the realm of peptide self-assembly, further highlighting that the underlying physicochemical properties are largely determined by the constituent components.

### 4.2 Controllable and data-efficient generation with SAPGEN

Traditional machine-learning algorithms, such as the GA baseline, show limited compliance with certain conditional constraints, as observed by their low hit rates for properties in our generation tasks. Under these conditions, further screening of the generated sequences may not substantially improve the constraint satisfaction. This limitation severely restricts their practical utility in real-world peptide design, where their intrinsic properties must be aligned with specific application environments. By conditioning on structured prompts, SAPGEN enables the generation of peptides that satisfy specified constraints. In contrast to conventional peptide generators, which require extensive task-specific data, SAPGEN demonstrates reliable controllable generation under severe data scarcity. This capability arises from leveraging large-scale peptide pretraining, which encodes transferable sequence-property priors, allowing subsequent fine-tuning to focus on aligning generative trajectories with target attributes rather than relearning sequence grammar.

### 4.3 Limitations and future directions

Although our proposed framework provides a promising approach for the predictive and generative design of self-assembling peptides, several aspects still warrant further improvement. First, the extreme scarcity of data still constrains the complexity and novelty of the generated peptides to some extent. Moreover, the biological activities and potential toxicities of the generated sequences have not been experimentally verified, limiting their immediate applicability.

In future, expanding the dataset by incorporating experimentally validated and structurally diverse peptides will further improve the robustness and interpretability of this model. Additionally, exploring more stable and interpretable generative architectures, such as integrating reinforcement learning or diffusion-based strategies (Luo *et al.* 2024, Cao *et al.* 2025) could further enhance the usability of the model in relevant domains and its potential for the cogeneration of multiple biological activities. Finally, combining computational predictions with experimental validation will enable a more comprehensive evaluation of the predictive accuracy and biological relevance.

## 5 Conclusion

In this study, we propose a deep learning framework to control the design of peptides with self-assembly capabilities tailored to their intrinsic properties of length, isoelectric point (pI), and hydrophobicity. By integrating large-scale pretraining with a conditional autoregressive architecture, our approach effectively captures the sequence-property relationships relevant to the self-assembly of short peptides. Coupled with machine learning-based prediction, the framework facilitates robust screening of candidate sequences, potentially reducing the reliance on costly molecular dynamics simulations or labour-intensive experiments in peptide discovery. Our results suggest that the generated peptides tend to exhibit distinct self-assembly behaviours, highlighting the potential of this framework for the tailored design of peptide-based materials for biomedical and bioengineering applications.

## Supplementary Material

btag744_Supplementary_Data
