# Supplementary material for: DeepSAP: an integrated generative and predictive deep-learning framework for controllable design of self-assembling peptides
Source: Bioinformatics. 2026 Oct 7;42(10):btag744. doi: 10.1093/bioinformatics/btag744 (PMC13682847; doi:10.1093/bioinformatics/btag744)
Supplement: Supplementary file 1 [file btag744_supplementary_data.docx]

**Supplementary Information**

**DeepSAP: An Integrated Generative and Predictive Deep-Learning Framework for Controllable Design of Self-Assembling Peptides**

Yuanming Song¹², Yuhan Wu¹², Yuqiu Jiang¹², Shoubo Zhao¹², Li He¹², Xiaoyuan Deng¹²*

¹ MOE Key Laboratory of Laser Life Science & Institute of Laser Life Science, College of Biophotonics, School of Optoelectronic Science and Engineering, South China Normal University, Guangzhou 510631, China

² Guangdong Provincial Key Laboratory of Laser Life Science, Guangzhou Key Laboratory of Spectral Analysis and Functional Probes, College of Biophotonics, School of Optoelectronic Science and Engineering, South China Normal University, Guangzhou 510631, China

*Corresponding author

Email address: xydeng12@126.com

**Supplementary Methods**

**S1. Peptide Feature Encoding**

S1.1 Calculation of handcrafted features

1.1.1 Amino Acid Composition (AAC)

The amino acid composition (AAC) feature represents the normalized frequency of each of the 20 standard amino acids in a peptide sequence.

For a sequence of length L, the AAC for amino acid i is defined as:

$$\begin{aligned} \text{AAC}_{\text{i}}=\frac{\text{n}_{\text{i}}}{L}, i\in\left\{ 1,2,\ldots,20 \right\}\#\left( S1 \right) \end{aligned}$$

where n_i_ is the number of occurrences of the i-th amino acid.The resulting feature vector has 20 dimensions, and the sum of all elements equals 1.

1.1.2 Composition of k-Spaced Amino Acid Group Pairs (CKSAAGP)

The CKSAAGP descriptor measures the frequency of amino acid group pairs separated by k residues, capturing short-range interaction patterns.

First, the 20 amino acids are classified into 5 physicochemical groups according to side-chain properties (e.g., hydrophobic, polar, charged, aromatic, etc.).Let G(x) denote the group of residue x. For a given spacing k, the pair frequency is defined as:

$$\begin{aligned} \mathrm{CKSAAGP}_{g_{i},g_{j}}^{(k)}=\frac{N_{g_{i},g_{j}}^{\left( k \right)}}{N^{\left( k \right)}}\#\left( S2 \right) \end{aligned}$$

where: $\text{N}_{\text{g}_{\text{i}}\text{g}_{\text{j}}}^{\text{(}\text{k}\text{)}}$ is the number of times a residue from group $\text{g}_{\text{i}}$ is followed by a residue from group $\text{g}_{\text{j}}$ with $\text{k}$ residues in between;$\text{N(k)=L-k-1}$ is the total number of possible pairs for spacing $\text{k}$; k∈{1,2,3}.

For 5 groups, there are 5×5=25 pair types.Thus, for k=1,2,3 the feature dimension is 3×25=75.

1.1.3 Pseudo Amino Acid Composition (PseAAC)

The pseudo amino acid composition incorporates sequence-order information by adding correlation factors between physicochemical properties of amino acids.

The feature vector includes both 20 amino acid frequencies and λ correlation terms:

$$\begin{aligned} \mathrm{PseAAC}=\left[ \text{f}_{1}, \text{f}_{2}, \text{f}_{3},\ldots,\text{f}_{20},\theta_{1},\theta_{2},\ldots,\theta_{\lambda} \right]\#\left( S3 \right) \end{aligned}$$

Where: $\text{f}_{\text{i}}\text{=}\frac{\text{n}_{\text{i}}\text{ }}{\text{L}}$ is the normalized frequency of the i-th amino acid; $\text{θ}_{\text{k}}$is the sequence-order correlation factor defined as:

$$\begin{aligned} \theta_{\text{k}}=\frac{\text{1}}{\text{L-k}}\sum_{\text{i=1}}^{\text{L-k}} \text{C(}\text{R}_{\text{i}}\text{,}\text{R}_{\text{i+k}}\text{)}\#\left( S4 \right) \end{aligned}$$

Here C(R_i_, R_i+k_) denotes the correlation between physicochemical properties (e.g., hydrophobicity, polarity, or side-chain volume) of residues R_i_ and R_i+k_.For this study, we set λ=2, leading to 20+3=23 dimensions (including a global normalization factor).

1.1.4 Binary Profile Feature (BPF)

The binary profile feature encodes each amino acid as a one-hot vector of length 21 (20 amino acids + 1 padding position).A peptide is represented as a position-specific binary matrix of size L×21.
For sequences shorter than 10 residues, zero-padding is applied; for longer sequences, only the first 10 residues are used. Formally, for position p and amino acid a:

$$\begin{aligned} \text{BPF}_{\text{p,a}}=\left\{ \begin{aligned} \text{1, if residue at position p is a} \\ \text{0, otherwise } \end{aligned} \right.\#\left( S5 \right) \end{aligned}$$

The flattened feature vector therefore has 10×21=210 dimensions.

S1.2 AAindex features

Physicochemical properties were extracted from the AAindex database (v9.2) [1]. XGBoost [2] was employed for feature analysis owing to its robustness and interpretability in feature evaluation. For each amino acid, 566 physicochemical indices were retrieved, and the values corresponding to all residues within a peptide were summed to generate a 566-dimensional feature vector representing each sequence. An XGBoost model (default parameters; scikit-learn 1.2.2 [3]) was subsequently applied to identify the ten most informative indices, including peptide linkage propensity (H BAEK050101, score = 0.09945), normalised frequency of isolated helix (H TANS770102, score = 0.0458), and side-chain conformation (H YANJ020101, score = 0.03999). The complete list of selected indices, together with their descriptions and importance scores, is provided in Supplementary Table S2. Each selected index contributes a scalar value per sequence. Following sequence padding, the 10-dimensional AAindex physicochemical features of each residue were concatenated to form a 100-dimensional vector for each peptide, and the residue-wise summed values were further calculated to obtain an additional 10-dimensional representation.

S1.3 Aggrescan3d (A3D) Features

We compiled 21,641 historical A3D computation records previously generated on our local workstation. Each record comprises an amino acid sequence and the corresponding per-residue A3D aggregation propensity scores, organized in tabular form (e.g., sequence, pos_1 … pos_10). The dataset encompasses peptides of varying lengths (typically 4–10 amino acids), with shorter sequences zero-padded at missing positions. A sequence-based predictor was subsequently developed by fine-tuning an ESM protein language model coupled with a regression head to learn the direct mapping from primary sequence to A3D scores. This strategy obviates the requirement for three-dimensional structural inputs—otherwise necessary in the original A3D pipeline—and thereby eliminates the computational bottleneck associated with large-scale structure prediction models such as ESMFold. The model achieved a mean squared error (MSE) of < 0.01 and an R² value of 0.982 on the held-out test set, demonstrating excellent concordance with the original A3D outputs. Notably, the proposed framework enables direct prediction from sequence alone, leading to substantial acceleration compared with conventional structure-dependent workflows.

**S2. Counterfactual analysis of SAPBRF**

To elucidate the decision boundaries of the SAPBRF model (Section 2.6) and to assess the impact of sequence mutations on self-assembly predictions, counterfactual analysis [4,5] was performed on the test-set sequences described in Section 2.1. In contrast to attention-based approaches that require Transformer architectures, counterfactual analysis is well suited to the random forest framework of SAPBRF, which relies on the feature-engineering strategy outlined in Section 2.6.

For each peptide of length L, 19 × L single-point mutant sequences were generated by substituting each residue with the remaining 19 standard amino acids. This controlled perturbation scheme enables direct attribution of functional changes to individual residues, while avoiding the combinatorial explosion associated with multi-site mutations [6]. For each mutation, the log-odds ratio (LOR) was calculated as:

$$\begin{aligned} \text{log\_odds\_ratio}_{\text{i,k}}\text{=}\text{log}_{\text{2}}\text{(}\frac{\text{mutate\_proba}_{\text{i,k}}}{\text{origin\_proba}}\text{)}\#\left( S6 \right) \end{aligned}$$

where ${\text{mutate}\text{\_}\text{proba}}_{\text{i}\text{,}\text{k}}$ denotes the positive-class probability predicted by SAPBRF after the k-th mutation at position i, and$\text{ }\text{origin}\text{\_}\text{proba}$ represents the corresponding probability for the original (wild-type) sequence. The average LOR at each position was then computed as:

$$\begin{aligned} \text{log\_odds\_ratio}_{\text{i}}\text{=}\frac{\text{1}}{\text{N}}\sum_{\text{k=1}}^{\text{N}} \text{log\_odds\_ratio}_{\text{i,k}}\#\left( S7 \right) \end{aligned}$$

where N denotes the number of mutations per position (typically 19). Each positional LOR was subsequently standardised to a Z-score:

$$\begin{aligned} \text{Zscore}_{\text{i}}\text{=}\frac{\text{log\_odds\_ratio}_{\text{i}}\text{-μ}}{\text{σ}}\#\left( S8 \right) \end{aligned}$$

where μ and σ represent the mean and standard deviation of the LOR values across the mutation batch. The results were visualised as sensitivity heatmaps (Fig. S2D). For a sequence of length L, the computational complexity of this analysis is O(L x N), where N is the number of mutated sequences, enabling its use for guiding sequence design and mechanistic analysis.

The counterfactual analysis results are presented in Fig. S2. The global mutation sensitivity—defined as the average LOR change across all amino acid substitutions—was 10.19% (Fig. S2E), indicating overall robustness to random single-point mutations. Site-specific analysis revealed heterogeneous positional effects (Fig. S2D): residues at position 6 were relatively tolerant to mutation (mean LOR = 0.05715), whereas mutations at positions 3–5 generally suppressed self-assembly predictions (mean LOR = −0.04218 and −0.04334, respectively). At the residue level, substitutions to proline (P) and glutamic acid (E) resulted in the largest decreases in predicted scores, whereas isoleucine (I) and leucine (L) produced the most pronounced increases (Fig. S2A–C).

Fig S1. Counterfactual Analysis results of peptides in test set

To further illustrate the practical utility of this approach, counterfactual analysis was applied to selected peptide sequences as case studies (Fig. S1). Six representative peptides were examined, including four self-assembling (positive) samples (Fig. S1I–III) and two non-self-assembling (negative) samples (Fig. S1IV–VI). Heatmaps were visualised using a red-to-blue colour scale (range: −1.5 to +1.5; Z-score normalised), representing the effect of single-site mutations on prediction outcomes. Positive samples typically exhibited spatially continuous red regions, predominantly located in the central or terminal segments of the sequence (e.g., FLVHSS (I) and LIAVGD (II)). In contrast, negative samples were characterised primarily by neutral (white) or inhibitory (blue) regions, without prominent mutational hotspots.

Fig S2. Statistical results of single-point mutation counterfactual analysis. (A) Sensitivity of original amino acids across positions. (B) Sensitivity of mutated amino acids across positions. (C) Heatmap of prediction change by position and mutated amino acid (AA). (D) Distribution of mutation sensitivity per position (log odds ratio). (E) Distribution of average probability change across positions.

**S3. Feature Contribution in SAPBRF model**

Furthermore, AAindex feature analysis identified H_BAEK050101, H_TANS770102 and H_YANJ020101 as the most influential descriptors (Fig. S3A–B, D). Figure S3A presents the top 20 AAindex features ranked by XGBoost importance, demonstrating that backbone linkage propensity contributes most substantially to the prediction. In the SHAP summary plot (Fig. S3B), H_BAEK050101 (linkage index) and H_YANJ020101 (side-chain conformation) exhibit the strongest positive effects on peptide self-assembly, followed by hydrophobicity-related indices (H_PONP800103 and H_CIDH920104). By contrast, the flexibility descriptor H_VINM940102 displays predominantly negative SHAP values, suggesting that rigid backbones, stable rotamer conformations and hydrophobic packing favour aggregation, whereas increased backbone mobility impedes this process. Figure S3D further indicates that RACS770103 (side-chain orientational preference) and H_BAEK050101 show the clearest separation between self-assembling and non-self-assembling peptides, with positive samples exhibiting significantly higher Z-scores. TANS770102, VINM940102 and ROBB760113 (information measure for loop regions) also demonstrate evident class discrimination, whereas the remaining features show substantial overlap between classes.

Fig S3. XGBoost analysis and performance comparison. (A) Top 20 AA Index (AAIndex) feature importances by XGBoost. (B) SHAP analysis results of top 20 AAIndex features, with high and low feature values. (C) ROC curves comparison for different models (AUC values: 0.9778, 0.8889, 0.9564, 0.8418,0.9606). (D) Standardized distribution of top 20 AAIndex features by label. Feature representation: f1:H BAEK050101, f2:H TANS770102, f3:H YANJ020101, f4:H VINM940102, f5:H JOND920101, f6: H PONP800103, f7: H CIDH920104, f8: H ROBB760113, f9: H QIAN880128, f10:H QIAN880121, f11:H MAXF760105, f12: H FUKS010111, f13: H ROBB760108, f14: H RACS770103, f15:H CORJ870105, f16: H KARP850101, f17:H MUNV940104, f18: H FINA910101, f19:H FINA910103, f20:H DAYM780201.

**S4. Characteristics of the ml-sap model and threshold selection**

The ml-sap model is an external predictive tool employed to estimate the self-assembly propensity of peptide sequences. During preliminary evaluation, its output scores were found to exhibit a pronounced bimodal distribution, clustering near 0 and 1 with few intermediate values. This pattern suggests poor calibration and probable overfitting to the original training dataset, leading to excessively confident predictions. Consequently, numerous peptides—including sequences unlikely to self-assemble—were assigned scores exceeding 0.9. To mitigate inflated false-positive rates and ensure more conservative assessments, a stringent threshold of 0.9 was adopted to define positive predictions in this study. This criterion facilitates more reliable comparisons across generation tasks and reduces the risk of systematic overestimation of self-assembly propensity.

**S5. Implement of CGMD in details**

To evaluate the self-assembly behaviour of the generated peptides at the melanoscale level, we introduced coarse-grained molecular dynamics (CGMD) simulations [7]. All molecular dynamics simulations were conducted using the GROMACS 2023.2 software package [8] with coarse-grained simulations based on the Martini 2.2P force field [9]. Initially, peptide sequences were converted into the corresponding coarse-grained models using the Martinize tool. Subsequently, 120 peptide molecules were randomly solvated in a 20 nm × 20 nm × 20 nm box with water at a density of approximately 1 g/cm3. Coarse polarisable water beads were used in the simulations. Following the system setup, simulations were performed in three stages: energy minimisation, equilibration, and production. The energy minimisation stage employed the steepest descent algorithm to eliminate unreasonable contacts in the initial conformation. The equilibration stage consisted of two steps under constant temperature and pressure, maintaining the system at 300 K and 1 bar. Temperature control was achieved using the V-rescale and Nosé–Hoover thermostat, whereas pressure control utilised the C-rescale and Berendsen barostat, with semi-isotropic coupling applied based on the system anisotropy. Production simulations were conducted in the NPT ensemble with a time step of 20 fs and total simulation time of 100 ns [10]. During simulations, van der Waals interactions were modified using a force-switch function, and electrostatic interactions were treated with the reaction-field method (dielectric constant ɛr = 2.5) [7][11]. To characterise the degree of self-assembly quantitatively, we adopted the aggregation propensity (AP) value, which was calculated as follows:

$$\begin{aligned} \text{AP}\text{=}\frac{\text{SASA}_{\text{initial}}}{\text{SASA}_{\text{final}}}\#\left( \text{S}\text{9} \right) \end{aligned}$$

where SASA_initial_ is the solvent accessible surface area of the peptide molecule in the initial minimization frame and SASA_final_ is the solvent-accessible surface area (SASA) of the final configuration of the simulation [12]. System visualisation and structural analysis were performed using VMD software [12].

**S6. Comparison Between the Original MD System and the Current MD Setup**

To ensure consistency between our molecular dynamics (MD) simulations and those reported in the reference study, we reproduced the aggregation propensity (AP) calculations for nine benchmark peptides using our simulation protocol and compared the results with the previously published AP values. The findings are summarizcsed in Supplementary Table S4. Across all peptides, the mean signed difference between the two protocols was 0.012, and the mean absolute deviation was 0.057, indicating close agreement in the resulting aggregation scores. Importantly, no systematic upward or downward shift was detected, and the maximum absolute deviation (approximately 0.11) remained within the expected numerical variation of short-timescale peptide MD simulations. In the absence of systematic bias and given the small magnitude of the deviations, the threshold of AP = 1.75 adopted in the original study was retained. This choice ensures direct comparability between datasets while remaining robust to minor numerical fluctuations across simulation systems. Adjusting the threshold based on the mean difference (1.75 + 0.012 ≈ 1.76) would produce a negligible effect and was therefore deemed unnecessary. Accordingly, AP = 1.75 remains a justified and reproducible cut-off for distinguishing assembling from non-assembling peptides within our MD framework.

**S7. Fine-tuning details for ProtGPT2 and ProtRL models**

For peptide generation, the genetic algorithm (GA) proposed in [10], fine-tuned ProtGPT2 [13], a pretrained protein language model, and ProtRL [15], a reinforcement learning-based peptide generation model, were used as baselines.

For ProtGPT2, the publicly available pretrained model was fine-tuned on the self-assembling peptide dataset using the official Hugging Face run_clm.py script for causal language modeling. Fine-tuning was performed with a learning rate of 1 × 10^−6^, with block size of 6.

ProtRL was fine-tuned following the SFT+GRPO workflow recommended by the original GitHub repository, using ProtGPT3 as the initial model. The original basic model ZymCTRL was not adopted because it was originally developed for enzyme protein generation and is less suited to the short peptide generation task studied here. Therefore, ProtGPT3 was used as the backbone recommended by the ProtRL implementation. For each optimization task, the model was trained for 300 reinforcement-learning iterations, with 256 peptide sequences generated per iteration and a training batch size of 4. Sequence generation was performed with a maximum length of 15, a temperature of 1.0, and a top-p value of 0.95. For each generated peptide, the reward was calculated from its self-assembly propensity predicted by SAPBRF and its target physicochemical properties, including sequence length, pI, and GRAVY. The length reward was defined using an exponential decay function based on the absolute deviation from the target length, while the pI reward followed a Gaussian function centred on the target pI. The GRAVY reward was set to 1 within the target interval and decayed exponentially with increasing distance outside the interval. The overall reward was calculated as 0.5RSAP + 2Rlength + 1.5RpI + 0.5RGRAVY and subsequently squared to sharpen the reward differences. No additional reward normalization was applied during training. The SAPBRF model used for reward calculation was kept fixed throughout the reinforcement-learning process.

**S8. Fine-tuning details of ProtBERT-LoRA and ESM2-LoRA models**

For predictive modeling, ML-SAP [10], a machine learning-based SAP predictor, and fine-tuned ProtBERT [15] and ESM2 [16], the pretrained protein language models, were used as baselines.

Both ProtBERT and ESM2 were fine-tuned using the same training, validation, and test splits as SAPBRF to ensure a fair comparison. The pretrained protein language models were initialized from their publicly available checkpoints and adapted for binary peptide self-assembly classification using Low-Rank Adaptation (LoRA). For each peptide sequence, amino acids were tokenized using the corresponding pretrained tokenizer. Individual residues were separated by whitespace before tokenization, and uncommon amino acids were replaced with "X". Sequences were padded or truncated to a maximum length of 18 residues. LoRA adapters were inserted into the attention projection layers of the transformer encoder. The LoRA configuration employed a rank of 4, a scaling factor (α) of 1, and a dropout rate of 0.3. During fine-tuning, only the LoRA parameters and the sequence classification head were optimized, while all pretrained backbone parameters remained frozen.

Training was performed using the Hugging Face Transformers Trainer with the AdamW optimizer. The learning rate was set to 5 × 10⁻⁴, the batch size was 256 for both training and evaluation, and models were trained for 20 epochs with a weight decay of 1 × 10⁻⁴. Model performance on the validation set was evaluated after each epoch, and the checkpoint achieving the highest weighted F1-score was retained for subsequent testing.

Performance was assessed using Accuracy (ACC), Precision, Recall (Sensitivity), Specificity (SP), weighted F1-score, and the Area Under the Receiver Operating Characteristic Curve (AUC).

**S9. Extended Coarse-Grained Molecular Dynamics Validation of Representative SAP Design Tasks**

To further evaluate the robustness and generalizability of the proposed peptide design workflow, we extended the scope of coarse-grained molecular dynamics (CGMD) validation from the two representative prompts presented in the main text to all ten representative SAP design tasks (Sa1–Sa10) listed in Table 2. For each design task, SAPGEN generated peptides under the corresponding constraints as described in Table 2. Subsequently, the generated candidate peptides were ranked using SAPBRF, and the top three candidates were selected for CGMD simulations following the same protocol described in Supplementary S5. This process involved the evaluation of 30 peptides, covering the full range of representative SAP generation tasks.

The results of the extended CGMD validation demonstrate that the proposed generation and screening workflow is robustly applicable to a diverse range of conditional generation tasks. Detailed results of the peptide simulation tests are summarized in Supplementary Table S7.

**S10. Performance Evaluation Metrics**

To evaluate the biochemical plausibility and diversity of the peptides generated by SAPGEN, we used the BLOSUM62 scoring matrix [14] and Levenshtein distance [18]. The BLOSUM62 matrix, a widely used substitution matrix derived from the alignment of evolutionarily related proteins, was used to quantify the similarity between generated and reference peptide sequences. The similarity score for the two sequences of length L was calculated as follows:

$$\begin{aligned} \text{BLOSUM62(}\text{S}_{\text{1}}\text{,}\text{S}_{\text{2}}\text{)=}\sum_{\text{i=1}}^{\text{L}} \text{M(}\text{S}_{\text{1}}\text{[i],}\text{S}_{\text{2}}\text{[i])}\#\left( S10 \right) \end{aligned}$$

where M (a, b) represents the substitution score for amino acids a and b in the BLOSUM62 matrix, reflecting the similarity between amino acid pairs.

The diversity within the generated sequences was evaluated by calculating the Levenshtein Distance. For two strings s = s1 s2...sm and t = t1 t2...tn, the computation is defined as follows:

$$\begin{aligned} \text{D(i,j)=}\left\{ \begin{aligned} \text{0, }\text{ }\text{ }\text{ }\text{ }\text{ }\text{if i=0 and j=0}\text{ } \\ \text{ }\text{i, }\text{ }\text{ }\text{ }\text{ }\text{ }\text{ }\text{ if j=0}\text{ } \\ \text{ }\text{j, }\text{ }\text{ }\text{ }\text{ }\text{ }\text{if j=0}\text{ } \\ \text{min}\left\{ \begin{aligned} \text{ }\text{D(i-1,}\text{ }\text{j)+1 }\text{ }\text{ }\text{ }\text{ } \\ \text{ }\text{D(i,}\text{ }\text{j-1)+1, }\text{ }\text{ }\text{ }\text{otherwise }\text{ }\text{ }\text{ } \\ \text{ }\text{D(i-1,}\text{ }\text{j-1)+[}\text{s}_{\text{i}}\text{≠}\text{t}_{\text{j}}\text{] }\text{ }\text{ }\text{ }\text{ } \end{aligned} \right. \end{aligned}\text{ } \right.\#\left( S11 \right) \end{aligned}$$

Here,

$$\begin{aligned} \text{[}\text{s}_{\text{i}}\text{≠}\text{t}_{\text{j}}\text{]=}\left\{ \begin{aligned} \text{0, if }\text{s}_{\text{i}}\text{=}\text{t}_{\text{j}} \\ \text{1, if }\text{s}_{\text{i}}\text{≠}\text{t}_{\text{j}} \end{aligned} \right.\#\left( S12 \right) \end{aligned}$$

To enable a comparison across lengths and obtain a standard measure in [0,1], we used the normalised Levenshtein distance, defined as:

$$\begin{aligned} \text{D}_{\text{norm}}\text{(s,t)=}\frac{\text{D(s,t)}}{\text{max(|s|,|t|)}}\#\left( S13 \right) \end{aligned}$$

Uniqueness was calculated as the proportion of unique sequences among all generated sequences, defined as:

$$Uniqueness=\frac{N_{unique}}{N_{generated}}\#(S14)$$

To evaluate the performance of our proposed predictor, we used traditional evaluation metrics commonly used in binary classification tasks. Accuracy (ACC), sensitivity (SN), specificity (SP), F1 score [19], and area under the curve (AUC) [20] were used to evaluate the classification performance, with the following computations:

$$\begin{aligned} \left\{ \text{ }\begin{aligned} ACC=\frac{TP+TN}{TP+TN+FP+FN}, \\ \text{SN=}\frac{\text{TP}}{\text{TP+FN}}\text{, }\text{ }\text{ } \\ \text{SP=}\frac{\text{TN}}{\text{TN+FP}}\text{, }\text{ }\text{ } \\ \text{F1=}\frac{\text{2·Precision·Recall}}{\text{Precision+Recall}}\text{,}\text{ }\text{ } \\ \text{AUC=}\int_{\text{0}}^{\text{1}} \text{ROC(t)dt.}\text{ }\text{ }\text{ }\text{ } \end{aligned} \right.\#\left( S15 \right) \end{aligned}$$

Here, TP, TN, FP, and FN represent true positives, true negatives, false positives, and false negatives, respectively, and ROC(t) denotes the receiver operating characteristic (ROC) curve value at threshold t.

**Supplementary Tables**

**Table S1** Selected peptide types and their quantities

| **Activity** | **Num** |
| --- | --- |
| Stimulator | 1384491 |
| Anti oxidative | 981113 |
| Regulatory | 904016 |
| Angiotensinase inhibitor | 872569 |
| Anti tuberculosis | 717299 |
| Coagulation and vascular | 715691 |
| Anti orthomyxoviridae | 664910 |
| Anticancer | 635926 |
| Immunomodulatory | 609016 |
| Antiviral | 565814 |
| Propeptide | 563092 |
| Immunological | 522049 |
| Anti hypertensive | 509979 |
| Signal peptide | 471381 |
| Anti inflamatory | 440170 |
| Blood brain barrier penetrating | 434574 |
| Anti gram positive | 416770 |
| Anti hepatitis B virus | 319069 |
| Anti diabetic type 2 | 314450 |
| Antimicrobial | 282438 |
| Tumor targeting | 276254 |
| Anti diabetic type 1 | 258762 |
| Immunoregulator | 252416 |
| Anti paramyxoviridae | 183737 |
| Neuropeptide | 178620 |
| Anti SARS-COV 2 | 159638 |
| Enzyme inhibitor | 146025 |
| Anti gram negative | 145004 |
| Anti dengue virus | 128407 |
| Tumor homing | 120564 |
| Anti herpesviridae | 109436 |
| Anti bunyaviridae | 70043 |
| Anti herpes simplex virus | 66997 |
| Potentiator | 58024 |
| Gluten immunogenic and celiac toxic | 53026 |
| Anti angiogenic | 47113 |
| Anti thrombotic | 47057 |
| Toxic | 46708 |
| Anti vaccinia | 39782 |
| Antiparasitic | 25112 |
| Anti influenza virus | 23090 |
| Drug delivery vehicle | 19383 |
| Anti amnesic | 19172 |
| Transit | 17334 |
| Antibacterial | 13788 |
| Angiotensin-converting enzyme inhibitors | 12458 |
| Inhibitor | 10669 |
| Anti diabetic | 9209 |
| Glucose | 8915 |
| Metabolic | 7287 |
| Anti coronavirus | 4433 |
| Allergen | 3930 |
| Anti mammalian cell | 3785 |
| Anti hantaviridae | 3679 |
| Other | 3558 |
| Anti filoviridae | 3383 |
| Anti sin nombre virus | 3318 |
| Anti hepatitis C virus | 3181 |
| Anti fungal | 2816 |
| Hormone | 2607 |
| Anti retroviridae | 2507 |
| Anti flaviviridae | 2477 |
| Neurotoxin | 2359 |
| Anti feline immunodeficiency virus | 2176 |
| Anti andes virus | 2071 |
| Anti HIV | 926 |
| Amyloidosis | 724 |
| Anti SARS-COV | 665 |
| Cell penetrating | 638 |
| Anuran defense | 581 |
| Anti human parainfluenza virus | 532 |
| Enzyme activator | 408 |
| Cytotoxic | 354 |
| Self assembly | 342 |
| Cell cell communication | 321 |
| Hemolytic | 314 |
| Pheromone | 290 |
| Activator | 217 |
| Anti candida | 121 |
| Anti poxviridae | 103 |
| Aggregation | 55 |

**Table S2** Top 10 Important AAIndex features and their descriptions

| **AAIndex Id** | **Description** | **Importance** |
| --- | --- | --- |
| H BAEK050101 | D Linker index | 0.0995 |
| H TANS770102 | D Normalized frequency of isolated helix | 0.0458 |
| H YANJ020101 | D Side-chain conformation by gaussian evolutionary method | 0.0400 |
| H VINM940102 | D Normalized flexibility parameters | 0.0364 |
| H JOND920101 | D Relative frequency of occurrence | 0.0358 |
| H PONP800103 | D Average gain ratio in surrounding hydrophobicity | 0.0319 |
| H CIDH920104 | D Normalized hydrophobicity scales for alpha/beta-proteins | 0.0298 |
| H ROBB760113 | D Information measure for loop | 0.0270 |
| H QIAN880128 | D Weights for coil at the window position of -5 | 0.0206 |
| H QIAN880121 | D Weights for beta-sheet at the window position of 1 | 0.0193 |

**Table S3** Model Hyperparameters

| **Category** | **Parameter** | **Symbol** | **Description** | **Value / Setting** |
| --- | --- | --- | --- | --- |
| **Model architecture** | Model type | – | Transformer-based autoregressive language model | Decoder-only |
|  | Number of layers | N_L_ | Transformer block layers | 12 |
|  | Hidden dimension | d_model | Token embedding and hidden size | 512 |
|  | Feed-forward dimension | d_f_ | Hidden size in FFN | 512 |
|  | Attention heads | h | Number of self-attention heads | 16 |
|  | Dropout rate | p | Applied to attention and feed-forward layers | 0.1 |
|  | Positional encoding | – | Type of positional representation | Learned sinusoidal |
|  | Vocabulary size | V | Total amino acid tokens + control tokens | 167 |
|  | Max sequence length | L_max_ | Maximum number of tokens per sequence | 15 |
| **Training configuration** | Optimizer | – | Optimization algorithm | AdamW |
|  | Learning rate | η | Initial learning rate | 1.00E-05 |
|  | Batch size | B | Sequences per batch | 256 |
|  | Epochs | E | Total training epochs | 20 |
|  | Weight decay | λ_w_ | L2 regularization | 1.00E-04 |
|  | Warmup ratio | – | Linear warm-up fraction | 0.05 |
|  | Loss function | – | Objective for generation | Cross-entropy |
| **Controlled generation** | Condition tokens | – | Encodes functional & physicochemical attributes | pI, Length, Task type |
|  | pI range encoding | – | Discrete bin of isoelectric point | [3.0,10] |
|  | Length control | – | Tokenized length constraint | 1-15 residues |
| **Implementation details** | Framework | – | Programming environment | Python 3.12, PyTorch 2.5.0 |
|  | Hardware | – | GPUs used for training | NVIDIA RTX 4090 (24 GB) |

**Table S4** Comparison of aggregation propensity (AP) values

| **Peptide** | **SASAi** | **SASAf** | **AP** | **APs** |
| --- | --- | --- | --- | --- |
| FGDAAGGNNF | 1425.10 | 726.010 | 1.96 | 1.88 |
| FATAAGGNNF | 1410.42 | 610.243 | 2.31 | 2.38 |
| FGDAAGGNTF | 1386.20 | 742.690 | 1.87 | 1.90 |
| FGDAAGGNTT | 1334.50 | 719.401 | 1.86 | 1.85 |
| FATAAGGNMF | 1403.43 | 638.045 | 2.19 | 2.19 |
| VWPPDP | 1759.95 | 954.066 | 1.84 | 1.73 |
| AKCPQP | 1509.02 | 988.106 | 1.52 | 1.63 |
| MKYKEE | 1827.39 | 1049.09 | 1.74 | 1.72 |
| VKYKEE | 1898.98 | 1065.37 | 1.78 | 1.70 |
| VKYKKE | 1854.24 | 1366.32 | 1.35 | 1.40 |

**Table S5** Distribution of SAP prompts in the original dataset (Top50)

| **Refined_Prompt** | **Num** |
| --- | --- |
| pI:5.5-6.0, Length:4, GRAVY:2.5-3.0 | 20 |
| pI:3.5-4.0, Length:6, GRAVY:1.0-1.5 | 16 |
| pI:5.5-6.0, Length:4, GRAVY:0.5-1.0 | 15 |
| pI:5.5-6.0, Length:4, GRAVY:1.0-1.5 | 15 |
| pI:5.5-6.0, Length:4, GRAVY:4.0-4.5 | 10 |
| pI:3.5-4.0, Length:6, GRAVY:1.5-2.0 | 9 |
| pI:5.5-6.0, Length:4, GRAVY:2.0-2.5 | 8 |
| pI:5.5-6.0, Length:6, GRAVY:2.0-2.5 | 8 |
| pI:3.5-4.0, Length:6, GRAVY:0.5-1.0 | 8 |
| pI:3.5-4.0, Length:6, GRAVY:2.0-2.5 | 7 |
| pI:5.5-6.0, Length:6, GRAVY:1.5-2.0 | 7 |
| pI:3.5-4.0, Length:6, GRAVY:0.0-0.5 | 6 |
| pI:5.5-6.0, Length:4, GRAVY:1.5-2.0 | 6 |
| pI:5.5-6.0, Length:4, GRAVY:3.5-4.0 | 5 |
| pI:8.0-8.5, Length:15, GRAVY:1.0-1.5 | 5 |
| pI:4.0-4.5, Length:6, GRAVY:0.0-0.5 | 4 |
| pI:5.5-6.0, Length:6, GRAVY:1.0-1.5 | 4 |
| pI:3.5-4.0, Length:3, GRAVY:1.0-1.5 | 4 |
| pI:5.5-6.0, Length:5, GRAVY:2.5-3.0 | 4 |
| pI:3.5-4.0, Length:5, GRAVY:0.0-0.5 | 4 |
| pI:8.0-8.5, Length:10, GRAVY:0.0-0.5 | 4 |
| pI:7.0-7.5, Length:4, GRAVY:1.5-2.0 | 4 |
| pI:8.0-8.5, Length:7, GRAVY:0.5-1.0 | 4 |
| pI:6.0-6.5, Length:5, GRAVY:0.0-0.5 | 3 |
| pI:5.5-6.0, Length:3, GRAVY:3.0-3.5 | 3 |
| pI:6.0-6.5, Length:7, GRAVY:-3.0--2.5 | 3 |
| pI:5.5-6.0, Length:6, GRAVY:2.5-3.0 | 3 |
| pI:5.5-6.0, Length:4, GRAVY:3.0-3.5 | 3 |
| pI:8.0-8.5, Length:7, GRAVY:-0.5-0.0 | 3 |
| pI:3.5-4.0, Length:10, GRAVY:0.0-0.5 | 3 |
| pI:5.5-6.0, Length:6, GRAVY:0.5-1.0 | 3 |
| pI:3.5-4.0, Length:4, GRAVY:0.0-0.5 | 3 |
| pI:8.0-8.5, Length:8, GRAVY:0.0-0.5 | 3 |
| pI:4.0-4.5, Length:6, GRAVY:0.5-1.0 | 2 |
| pI:5.5-6.0, Length:5, GRAVY:1.5-2.0 | 2 |
| pI:8.0-8.5, Length:10, GRAVY:-0.5-0.0 | 2 |
| pI:6.0-6.5, Length:4, GRAVY:-1.5--1.0 | 2 |
| pI:5.5-6.0, Length:4, GRAVY:0.0-0.5 | 2 |
| pI:5.5-6.0, Length:5, GRAVY:1.0-1.5 | 2 |
| pI:8.0-8.5, Length:11, GRAVY:-0.5-0.0 | 2 |
| pI:8.0-8.5, Length:11, GRAVY:-1.0--0.5 | 2 |
| pI:8.0-8.5, Length:10, GRAVY:0.5-1.0 | 2 |
| pI:3.5-4.0, Length:10, GRAVY:0.5-1.0 | 2 |
| pI:4.0-4.5, Length:6, GRAVY:1.0-1.5 | 2 |
| pI:3.5-4.0, Length:3, GRAVY:-1.0--0.5 | 2 |
| pI:5.0-5.5, Length:9, GRAVY:0.5-1.0 | 2 |
| pI:3.5-4.0, Length:5, GRAVY:1.5-2.0 | 2 |
| pI:8.0-8.5, Length:8, GRAVY:2.0-2.5 | 2 |
| pI:3.5-4.0, Length:4, GRAVY:1.5-2.0 | 2 |

**Table S6** CGMD validation results for the top-ranked peptides generated under unseen physicochemical constraints.

| **Peptide** | **SASAi** | **SASAf** | **AP** | **SAPBRF score** |
| --- | --- | --- | --- | --- |
| HCFHFNYLWN | 2014.033 | 873.145 | 2.306642 | 0.79487 |
| HCFQFHFYYN | 2023.174 | 850.374 | 2.379158 | 0.792031 |
| HCFFHFQFNW | 1982.11 | 879.786 | 2.252946 | 0.774529 |
| CCNFYYHYYF | 1882.005 | 815.77 | 2.307029 | 0.767733 |
| HFHFFFQFNW | 2061.469 | 826.402 | 2.494511 | 0.765523 |
| WCWHFWYY | 1753.058 | 726.934 | 2.411578 | 0.752565 |
| WWVWHWYC | 1992.285 | 872.098 | 2.284474 | 0.742788 |
| WWFHFHCW | 1990.232 | 928.958 | 2.142435 | 0.722565 |
| WHFHFYLY | 2057.134 | 861.94 | 2.386632 | 0.721714 |
| WWFHFHYF | 1986.317 | 839.25 | 2.366776288 | 0.73146 |

**Table S7** CGMD validation results of the top-ranked peptides generated for the representative SAP design tasks (Sa1–Sa10).

| **Task** | **Peptide sequence** | **SASA init** | **SASA final** | **AP score** |
| --- | --- | --- | --- | --- |
| Sa1 | LAVF | 1391.166 | 732.358 | 1.8996 |
|  | FVVG | 1426.556 | 669.099 | 2.1321 |
|  | VVFY | 1659.616 | 899.267 | 1.8455 |
| Sa2 | GYLF | 1600.173 | 711.85 | 2.2479 |
|  | YSIY | 1596.589 | 679.215 | 2.3506 |
|  | FWYF | 1924.872 | 741.429 | 2.5962 |
| Sa3 | LTLIYD | 1537.215 | 784.569 | 1.9593 |
|  | SFFIFD | 1742.757 | 749.322 | 2.3258 |
|  | LVLVYD | 1563.668 | 854.938 | 1.829 |
| Sa4 | STFIID | 1566.184 | 726.192 | 2.1567 |
|  | DVLIIN | 1521.682 | 828.084 | 1.8376 |
|  | ATIIIE | 1393.104 | 787.957 | 1.768 |
| Sa5 | GGGFVLVAGVVAAKK | 1229.478 | 741.681 | 1.6577 |
|  | IKVAVVGAVGAAGKG | 1150.474 | 704.11 | 1.6339 |
|  | KKKKKLVLLLLLFFF | 1736.131 | 991.684 | 1.7507 |
| Sa6 | CKFKFKFQFC | 1692.926 | 907.851 | 1.8648 |
|  | CKFKFKFQFL | 1765.177 | 904.999 | 1.9505 |
|  | KIKIYFQLFN | 1696.221 | 796.12 | 2.1306 |
| Sa7 | AYVYY | 1342.399 | 592.879 | 2.2642 |
|  | YYVYY | 1715.532 | 735.62 | 2.3321 |
|  | FYFYY | 1812.416 | 729.219 | 2.4854 |
| Sa8 | QYQYQYQ | 2089.9 | 841.001 | 2.485 |
|  | NYNYNYN | 1655.434 | 722.055 | 2.2927 |
|  | QYKYDYN | 1676.925 | 805.75 | 2.0812 |
| Sa9 | GGGGGGGGGGHG | 842.964 | 538.243 | 1.5661 |
|  | WWHHFHFHFWFF | 1598.286 | 786.846 | 2.0313 |
|  | GYYYYAYYHFGF | 1416.588 | 688.727 | 2.0568 |
| Sa10 | FHFFFY | 1946.205 | 888.134 | 2.1913 |
|  | LHFFHF | 1760.263 | 772.626 | 2.2783 |
|  | LVHLHF | 1642.843 | 748.212 | 2.1957 |

**Reference**

[1] Kawashima S, Kanehisa M. AAindex: amino acid index database. Nucleic Acids Res 2000; 28:374.
[2] Chen T, Guestrin C. XGBoost: a scalable tree boosting system. In: Proceedings of the 22nd ACM SIGKDD International Conference on Knowledge Discovery and Data Mining. 2016, 785–94.
[3] Pedregosa F, Varoquaux G, Gramfort A et al. Scikit-learn: machine learning in Python. J Mach Learn Res 2011; 12:2825–30.
[4] Pearl J. Causal inference in statistics: an overview. Stat Surv 2016; 3:96–146.
[5] Wachter S, Mittelstadt B, Russell C. Counterfactual explanations without opening the black box: automated decisions and the GDPR. Harv J Law Technol 2017; 31:841–87.
[6] Schreiber G, Fersht AR. Rapid, electrostatically assisted association of proteins. Nat Struct Biol 1996; 3:427–31.
[7] Frederix PWJM, Ulijn RV, Hunt NT et al. Virtual screening for dipeptide aggregation: toward predictive tools for peptide self-assembly. J Phys Chem Lett 2011; 2:2380–4.
[8] Abraham MJ, Murtola T, Schulz R et al. GROMACS: high performance molecular simulations through multi-level parallelism from laptops to supercomputers. SoftwareX 2015; 1–2:19–25.
[9] Souza PCT, Alessandri R, Barnoud J et al. Martini 3: a general purpose force field for coarse-grained molecular dynamics. Nat Methods 2021; 18:382–8.
[10] Njirjak M, Žužić L, Babić M et al. Reshaping the discovery of self-assembling peptides with generative AI guided by hybrid deep learning. Nat Mach Intell 2024.
[11] Frederix PWJM, Scott GG, Abul-Hajjia YM et al. Exploring the sequence space for (tri)peptide self-assembly to design and discover new hydrogels. Nat Chem 2015; 7:30–7.
[12] Humphrey W, Dalke A, Schulten K. VMD: visual molecular dynamics. J Mol Graph 1996; 14:33–8.
[13] Ferruz N, Schmidt S, Höcker B. ProtGPT2 is a deep unsupervised language model for protein design. Nat Commun 2022; 13:4348.
[14] Stocco, F., Artigues-Lleixa, M., Hunklinger, A., Widatalla, T., Güell, M., & Ferruz, N. (2025). Guiding generative protein language models with reinforcement learning. arXiv. <https://doi.org/10.48550/arXiv.2412.12979.>
[15] Elnaggar A et al. ProtTrans: towards cracking the language of life’s code through self-supervised deep learning and high performance computing. IEEE Trans Pattern Anal Mach Intell 2021; 44:7112–27.
[16] Lin, Z., Akin, H., Rao, R., et al, Fazel-Zarandi, M., Sercu, T., Candido, S., & Rives, A. (2023). Evolutionary-scale prediction of atomic-level protein structure with a language model. Science, 379 (6637), 1123–1130. https://doi.org/10.1126/science.ade2574.
[17] Henikoff S, Henikoff JG. Amino acid substitution matrices from protein blocks. Proc Natl Acad Sci USA 1992; 89:10915–19.
[18] Levenshtein VI. Binary codes capable of correcting deletions, insertions and reversals. Sov Phys Dokl 1966; 10:707–10.
[19] Powers DMW. Evaluation: from precision, recall and F-measure to ROC, informedness, markedness and correlation. J Mach Learn Technol 2011; 2:37–63.
[20] Hanley JA, McNeil BJ. The meaning and use of the area under a receiver operating characteristic (ROC) curve. Radiology 1982; 143:29–36.
